# Formal reasoning about systems biology using theorem proving

**DOI:** 10.1371/journal.pone.0180179

**Published:** 2017-07-03

**Authors:** Adnan Rashid, Osman Hasan, Umair Siddique, Sofiène Tahar

**Affiliations:** 1 School of Electrical Engineering and Computer Science, National University of Sciences and Technology, Islamabad, Pakistan; 2 Department of Electrical and Computer Engineering, Concordia University, Montreal, QC, Canada; University of the West of England, UNITED KINGDOM

## Abstract

System biology provides the basis to understand the behavioral properties of complex biological organisms at different levels of abstraction. Traditionally, analysing systems biology based models of various diseases have been carried out by paper-and-pencil based proofs and simulations. However, these methods cannot provide an accurate analysis, which is a serious drawback for the safety-critical domain of human medicine. In order to overcome these limitations, we propose a framework to formally analyze biological networks and pathways. In particular, we formalize the notion of reaction kinetics in higher-order logic and formally verify some of the commonly used reaction based models of biological networks using the HOL Light theorem prover. Furthermore, we have ported our earlier formalization of Zsyntax, i.e., a deductive language for reasoning about biological networks and pathways, from HOL4 to the HOL Light theorem prover to make it compatible with the above-mentioned formalization of reaction kinetics. To illustrate the usefulness of the proposed framework, we present the formal analysis of three case studies, i.e., the pathway leading to TP53 Phosphorylation, the pathway leading to the death of cancer stem cells and the tumor growth based on cancer stem cells, which is used for the prognosis and future drug designs to treat cancer patients.

## Introduction

The discovery and design of effective drugs for infectious and chronic biological diseases, like cancer and cerebral malaria, require a deep understanding of behaviorial and structural characteristics of underlying biological entities (e.g., cells, molecules and enzymes). Traditional approaches, which rely on verbal and personal intuitions without concrete logical explanations of biological phenomena, often fail to provide a complete understanding of the behavior of such diseases, mainly due to the complex interactions of molecules connected through a chain of reactions. *Systems biology* [1] overcomes these limitations by integrating mathematical modeling and high-speed computing machines in the understanding of biological processes and thus provides the ability to predict the effect of potential drugs for the treatment of chronic diseases. System biology is widely used to model the biological processes as pathways or networks. Some of the examples are signaling pathways and protein-protein interaction networks [2]. These biological networks such as gene regulatory networks (GRNs) or biological regulatory networks (BRNs) [3], are analysed using the principles of molecular biology. This analysis, in turn, plays an important role for the investigation of the treatment of various human infectious diseases as well as future drug design targets. For example, the BRNs analysis has been recently used for the prediction of treatment decisions for sepsis patients [4].

Traditionally, biologists analyze biological organisms (or different diseases) using wet-lab experiments [5, 6]. These experiments cannot provide reliable analysis due to their inability to accurately characterize the complex biological processes in an experimental setting. Moreover, the experiments take a long execution time and often require an expensive experimental setup. One of the other techniques used for the deduction of molecular reactions is the paper-and-pencil proof method (e.g. Boolean modeling [7] or kinetic logic [8]). But the manual proofs in paper-and-pencil proof methods, become quite tedious for large systems, where several hundred proof steps are required in order to calculate the unknown parameters, thus prone to human error. Other alternatives for analyzing system biology problems include computer-based techniques (e.g. Petri nets [9] and model checking [10]). Petri net is a graph based technique [11] for analyzing system properties. In model checking, a system is modeled in the form of state-space or automata and the intended properties of the system are verified in a model checker by a rigorous state exploration of the system model. Theorem proving [12] is another formal methods technique that is widely used for the verification of the physical systems but has been rarely used for analyzing system biology related problems. In theorem proving, a computer-based mathematical model of the given system is constructed and then deductive reasoning is used for the verification of its intended properties. A prerequisite for conducting the formal analysis of a system is to formalize the mathematical or logical foundations that are required to model the system in an appropriate logic.

*Zsyntax* [13] is a recently proposed formal language that supports the modeling of any biological process and presents an analogy between a biological process and the logical deduction. It has some pre-defined operators and inference rules that are used for the logical deductions about a biological process. These operators and inference rules have been designed in such a way that they are easily understandable by the biologists, making Zsyntax a biologist-centered formalism, which is the main strength of this language. However, Zsyntax does not support specifying the temporal information associated with biological processes. *Reaction kinetics* [14], on the other hand, caters for this limitation by providing the basis to understand the time evolution of molecular populations involved in a biological network. This approach is based on the set of first-order ordinary differential equations (ODEs) also called *reaction rate equations* (RREs). Most of these equations are non-linear in nature and difficult to analyze but provide very useful insights for prognosis and drug predictions. Traditionally, the manual paper-and-pencil technique is used to reason logically about biological processes, which are expressed in Zsyntax. Similarly, the analysis of RREs is performed by either paper-and-pencil based proofs or numerical simulation. However, both methods suffer from the inherent incompleteness of numerical methods and error-proneness of manual proofs. We believe that these issues cannot be ignored considering the critical nature of this analysis due to the involvement of human lives. Moreover, biological experiments based on erroneous parameters, derived by the above-mentioned approaches may also result in the loss of time and money, due to the slow nature of wet-lab experiments and the cost associated with the chemicals and measurement equipment.

In this paper, we propose to develop a formal reasoning support for system biology to analyze complex biological systems within the sound core of a theorem prover and thus provide accurate analysis results in this safety-critical domain. By formal reasoning support, we mean to develop a set of generic mathematical models and definitions, a process that is usually termed as formalization, of commonly used notions of system biology using an appropriate logic and ascertain their properties as formally verified theorems in a theorem prover, which is a verification tool based on deductive reasoning. These formalized definitions and formally verified theorems can then in turn be used to develop formal models of real-world system biology problems and thus verify their corresponding properties accurately within the sound core of a theorem prover. The use of logic in modeling and a theorem prover in the verification leads to the accuracy of the analysis results, which cannot be ascertained by other computational approaches. In our recent work [15], we developed a formal deduction framework for reasoning about molecular reactions by formalizing the Zsyntax language in the HOL4 theorem prover [16]. In particular, we formalized the logical operators and inference rules of Zsyntax in higher-order logic. We then built upon these formal definitions to verify two key behavioral properties of Zsyntax based molecular pathways [17, 18]. However, it was not possible to reason about biological models based on reaction kinetics due to the unavailability of the formal notions of reaction rate equations (a set of coupled differential equations) in higher-order logic. In order to broaden the horizons of formal reasoning about system biology, this paper presents a formalization of reaction kinetics along with the development of formal models of generic biological pathways without the restriction on the number of molecules and corresponding interconnections. Furthermore, we formalize the transformation, which is used to convert biological reactions into a set of coupled differential equations. This step requires multivariate calculus (e.g., vector derivative, matrices, etc.) formalization in higher-order logic, which is not available in HOL4 and therefore we have chosen to leverage upon the rich multivariable libraries of the HOL Light theorem prover [19] to formalize the above mentioned notions and verify the reactions kinetics of some generic molecular reactions. To make the formalization of Zsyntax [15] consistent with the formalization of reaction kinetics in HOL Light, as part of our current work, we ported all of the HOL4 formalization of Zsyntax to HOL Light. In order to illustrate the usefulness and effectiveness of our formalization, we present the formal analysis of a molecular reaction representing the TP53 Phosphorylation [13], a molecular reaction of pathway leading to the death of cancer stem cells (CSC) and the analysis of tumor growth based on the CSC [20].

## Related work

In the last few decades, various modeling formalisms of computer science have been widely used in system biology. We briefly outline here the applications of computational modeling and analysis approaches in system biology, where the main idea is to transform a biological model into a computer program.

Process algebra (PA) [21] provides an expressive framework to formally specify the communication and interactions of concurrent processes without ambiguities. Biological systems can be considered as concurrent processes and thus process algebra can be used to model biological entities [22]. Some recent work in this area includes the formalizations of molecular biology based on *K*-Calculus [23] and *π*-Calculus [24]. The main tools that support PA in biology are sCCP [25], BioShape [26] and Bio-PEPA [27]. Even though PA based biological modeling provides sound foundations, it may be quite difficult and cumbersome for working biologists to understand these notations [28, 29].

Rule-based modeling offers a flexible and simple framework to model various biochemical species in a textual or graphical format. This allows biologists to perform the quantitative analysis [30, 31] of complex biological systems and predict important underlying behaviors. Some of the main rule-based modeling tools are BioNetGen [30], Kappa [32] and BIOCHAM [33]. These tools are mainly based on rewriting and model transformation rules along with the integration with model checking tools and numerical solvers. However, these integrations are usually not checked for correctness (for example by an independent proof assistant), which may lead to inconsistencies [34].

Boolean networks [35] are used to characterize the dynamics of gene-regulatory networks by limiting the behavior or genes by either a truth state or false state. Some of the major tools that support the Boolean modeling of biological systems are BoolNet [36], BNS [37] and GINsim [38]. The discrete nature of Boolean networks does not allow us to capture continuous biological evolutions, which are usually represented by differential equations.

Model checking has shown very promising results in many applications of molecular biology [39–42]. Hybrid systems theory [43] extends the state-based discrete representation of traditional model checking with a continuous dynamics (described in terms ODEs) in each state. Some of the recently developed tools that support the hybrid modeling of biological systems are S-TaLiRo [44], Breach toolbox [45] and dReach [46]. Recently, Petri nets have been widely used to model biological networks [47, 48] and some of the important associated tools include Snoopy [49] and GreatSPN [50]. However, the graph or state based nature of the models in these methods only allow the description of some specific areas of molecular biology [13, 51]. Moreover, the model checking technique has an inherent state-space explosion problem [52], which makes it only applicable to the biological entities that can acquire a small set of possible levels and thus limits its scope by restricting its usage on larger systems.

In a system analysis based on theorem proving, we need to formalize the mathematical or logical foundations required to model and analyze that system in an appropriate logic. Several attempts have been made to formalize the foundations of molecular biology. The first attempt at some basic axiomatization dates back to 1937 [53]. *Zanardo et al.* [54] and *Rizzotti et al.* [55] have also done some efforts towards the formalization of biology. But all these formalizations are paper-and-pencil based and have not been utilized to formally reason about molecular biology problems within a theorem prover. In our recent work [15], we developed a formal deduction framework for reasoning about molecular reactions by formalizing the Zsyntax language in the HOL4 theorem prover [16]. However, a major limitation of this work is that it cannot cater for the temporal information associated with biological processes and, hence, does not support modeling the time evolution of molecular populations involved in a biological network, which is of a dire need when studying the dynamics of a biological system. *Reaction kinetics* [14] provide the basis to understand the time evolution of molecular populations involved in a biological network. To overcome the limitation of the work presented by *Sohaib et al.* [15], we provide the formalization of reaction kinetics in higher-order logic and in turn extend the formal reasoning about system biology.

## Higher-order-logic theorem proving and HOL Light theorem prover

In this section, we provide a brief introduction to the higher-order-logic theorem proving and HOL Light theorem prover.

### Higher-order-logic theorem proving

Theorem proving involves the construction of mathematical proofs by a computer program using axioms and hypothesis. Theorem proving systems (theorem provers) are widely used for the verification of hardware and software systems [56, 57] and the formalization (or mathematical modeling) of classical mathematics [58–60]. For example, hardware designers can prove different properties of a digital circuit by using some predicates to model the circuits model. Similarly, a mathematician can prove the transitivity property for real numbers using the axioms of real number theory. These mathematical theorems are expressed in logic, which can be a propositional, first-order or higher-order logic based on the expressibility requirement.

Based on the decidability or undecidability of the underlying logic, theorem proving can be done automatically or interactively. Propositional logic is decidable and thus the sentences expressed in this logic can be automatically verified using a computer program whereas higher-order logic is undecidable and thus theorems about sentences, expressed in higher-order logic, have to be verified by providing user guidance in an interactive manner.

A theorem prover is a software for deductive reasoning in a sound environment. For example, a theorem prover does not allow us to conclude that “$\frac{x}{x}=1$” unless it is first proved or assumed that *x* ≠ 0. This is achieved by defining a precise syntax of the mathematical sentences that can be input in the software. Moreover, every theorem prover comes with a set of axioms and inference rules which are the only ways to prove a sentence correct. This purely deductive aspect provides the guarantee that every sentence proved in the system is actually true.

#### HOL Light theorem prover

HOL Light [19] is an interactive theorem prover used for the constructions of proofs in higher-order logic. The logic in HOL Light is represented in meta language (ML), which is a strongly-typed functional programming language [61]. A theorem is a formalized statement that may be an axiom or could be deduced from already verified theorems by an inference rule. Soundness is assured as every new theorem must be verified by applying the basic axioms and primitive inference rules or any other previously verified theorems/inference rules. A HOL Light theory is a collection of valid HOL Light types, axioms, constants, definitions and theorems, and is usually stored as an ML file in computers. Users interacting with HOL Light can reload a theory and utilize the corresponding definitions and theorems right away. Various mathematical foundations have been formalized and stored in HOL Light in the form of theories by the HOL Light users. HOL Light theories are organized in a hierarchical fashion and child theories can inherit the types, constants, definitions and theorems of the parent theories. The HOL Light theorem prover provides an extensive support of theorems regarding Boolean variables, arithmetics, real numbers, transcendental functions, lists and multivariate analysis in the form of theories which are extensively used in our formalization. The proofs in HOL Light are based on the concept of tactics which break proof goals into simple subgoals. There are many automatic proof procedures and proof assistants [62] available in HOL Light, which help the user in concluding a proof more efficiently.

## Proposed framework

**Fig 1:**
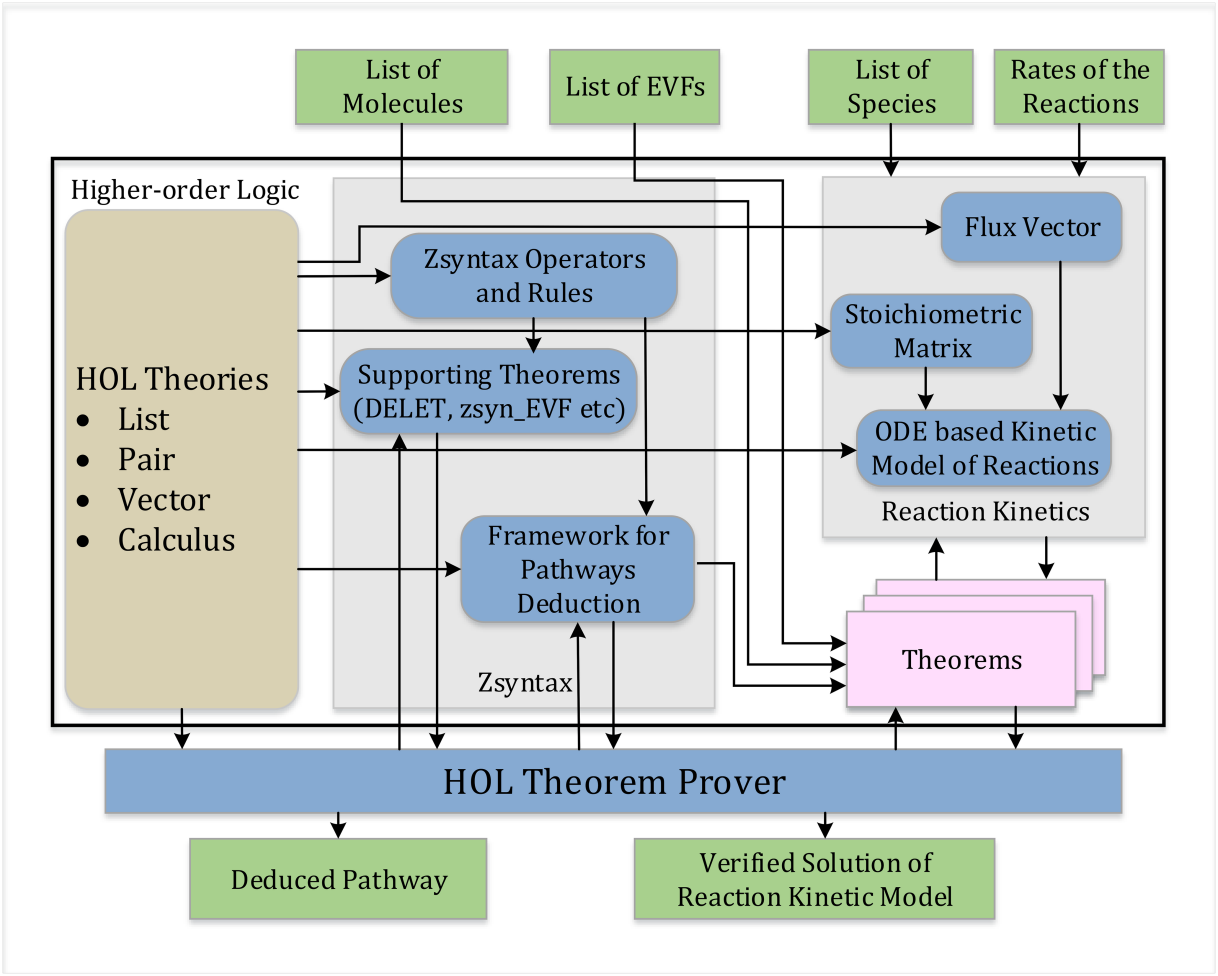
Proposed framework.

The proposed theorem proving based formal reasoning framework for system biology, depicted in Fig 1, allows the formal deduction of the complete pathway from any given time instance and model and analyze the ordinary differential equations (ODEs) corresponding to a kinetic model for any molecular reaction. For this purpose, the framework builds upon existing higher-order-logic formalizations of Lists, Pairs, Vectors, and Calculus.

The two main rectangles in the higher-order logic block present the foundational formalizations developed to facilitate the formal reasoning about the Zsyntax based pathway deduction and the reaction kinetics. In order to perform the Zsyntax based molecular pathway deduction, we first formalize the functions representing the logical operators and inference rules of Zsyntax in higher-order logic and verify some supporting theorems from this formalization. This formalization can then be used along with a list of molecules and a list of *Empirically Valid Formulae* (EVFs) to formally deduce the pathway for the given list of molecules and provide the result as a formally verified theorem using HOL Light. Similarly, we have formalized the flux vectors and stoichiometric matrices in higher-order-logic. These foundations can be used along with a given list of species and the rate of the reactions to develop a corresponding ODEs based kinetic reactions model. The solution to this ODE can then be formally verified as a theorem by building upon existing formalizations of Calculus theories.

The distinguishing characteristics of the proposed framework include the usage of deductive reasoning to derive the deduced pathways and solutions of the reaction kinetic models. Thus, all theorems are guaranteed to be correct and explicitly contain all required assumptions.

## Results

### Formalization of Zsyntax

Zsyntax [13] is a formal language which exploits the analogy between biological processes and logical deduction. Some of its key features are that: 1) it enables us to represent molecular reactions in a mathematical rigorous way; 2) it is of heuristic nature, i.e., if the initialization data and the conclusion of a reaction is known, then it allows us to deduce the missing data based on the initialization data; and 3) it possesses computer implementable semantics. Zsyntax has three operators namely *Z-Interaction*, *Z-Conjunction* and *Z-Conditional* that are used to represents different phenomenon in a biological process. These are the atomic formulas residing in the core of Zsyntax. Z-Interaction (*) represents the reaction or interaction of two molecules. In biological reactions, the Z-interaction operation is not associative. i.e., in a reaction having three molecules namely A, B and C, the operation (*A* ∗ *B*) ∗ *C* is not equal to *A* ∗ (*B* ∗ *C*). *Z-Conjunction (&)* is used to form the aggregate of the molecules participating in the biological process. These molecules can be same or different. Unlike the Z-Interaction operator, the Z-Conjunction is fully associative. *Z-Conditional (→)* is used to represent a path from *A* to *B* when condition *C* becomes true, i.e., *A* → *B* if there is a *C* allowing it. To apply the above-mentioned operators on a biological process, Zsyntax provides four inference rules that are used for the deduction of the outcomes of the biological reactions. These inference rules are given in Table 1.

**Table 1:** Zsyntax inference rules.

Inference Rules	Definition
Elimination of Z-conditional(→E)	if C ⊢ (A → B) and (D ⊢ A) then (C & D ⊢ B)
Introduction of Z-conditional(→I)	C & A ⊢ B then C ⊢ (A→B)
Elimination of Z-conjunction(& E)	C ⊢ (A & B) then (C ⊢ A) and (C ⊢ B)
Introduction of Z-conjunction(& I)	(C ⊢ A) and (D ⊢ B) then (C & D) ⊢ (A & B)

Zsyntax also utilizes the EVFs which are the empirical formulas validated in the lab and are basically the non-logical axioms of molecular biology. A biological reaction can be mapped and then these above-mentioned Zsyntax operators and inference rules are used to derive the final outcome of the reaction as shown in [13].

We start our formalization of Zsyntax, by formalizing the molecule as a variable of arbitrary data type (*α*) [18]. Z-Interaction is represented by a list of molecules (*α* *list*), which is a molecular reaction among the elements of the list. This (*α* list) may contain only a single element or it can have multiple elements. We model the Z-Conjunction operator as a list of list of molecules ((*α* *list*) *list*), which represents a collection of non-reacting molecules. Using this data type, we can apply the Z-Conjunction operator between individual molecules (a list with a single element), or between multiple interacting molecules (a list with multiple elements). Thus, based on our datatype, Z-Conjunction is a list of Z-interactions for both of these cases, i.e., individual molecules or multiple interacting molecules. So, overall, Z-conjunction acts as a set of Z-interaction. When a new set of molecules is generated based on the EVFs available for a reaction, the status of the molecules is updated using the Z-Conditional operator. We model each EVF as a pair of data type (*α* *list* # *α* *list* *list*) where the first element of the pair is a list of the molecules represented by data type (*α* *list*) and are actually the reacting molecules, whereas, the second element is a list of list of molecules ((*α* *list*) *list*), which represents a set of molecules that are obtained as a result of the reaction between the molecules of the first element of the pair and thus act as a set of Z-Interactions. A collection of EVFs is formalized using the data type ((*α* *list* # *α* *list* *list*) *list*), which is a list of EVFs.

**Fig 2:**
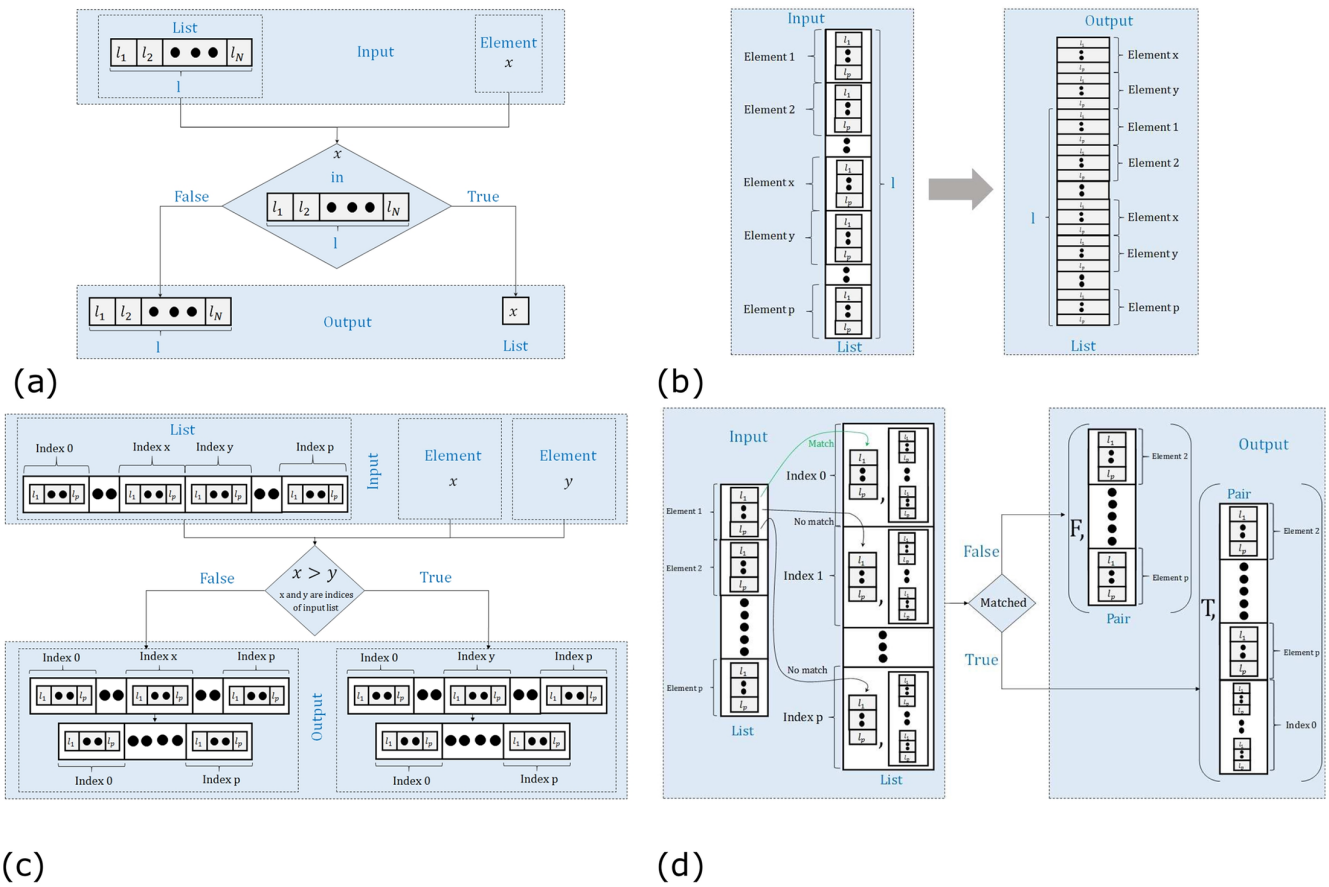
Graphical depiction of formalization of Zsyntax. (a) Elimination of the Z-Conjunction Rule (zsyn_conjun_elimin) (b) Introduction of Z-Conjunction (zsyn_conjun_intro) (c) Reactants Deletion (zsyn_delet) (d) EVF Matching (zsyn_EVF).

Next, we formalize the inference rules using higher-order logic. The inference rule named elimination of the Z-Conditional (→E) is equivalent to the Modus Ponens (the elimination of implication rule) law of propositional logic. Similarly, we can infer introduction of Z-Conditional (→I) rule from the existing rules of the propositional logic present in a theorem prover. Thus, both of these rules can be handled by the simplification and rewriting rules of the theorem prover and we do not need to define new rules for handling these inference rules. To check the presence of a particular molecule in an aggregate of some inferred molecules, the elimination of the Z-Conjunction (& E) rule is used. We apply it at the end of the biological reaction to check whether the product of the reaction is the desired molecule or not. We formalized this rule by a function (Table 2: zsyn_conjun_elimin), which accepts a list l and an element x and checks if x is present in this list. If the condition is true, it returns the given element x as a single element of that list l. Otherwise, it returns the list l as is, as shown in Fig 2a.

**Table 2:** Definitions of Zsyntax formalization.

Name	Formalized Form	Description
Elimination of Z-Conjunction Rule	⊢ ∀ l x. **zsyn_conjun_elimin** l x = **if** MEM x l **then** [x] else l	MEM x l: True if x is a member of list l
Introduction of Z-Conjunction and Z-Interaction	⊢ ∀ l x y. **zsyn_conjun_intro** l x y = CONS (FLAT [EL x l; EL y l]) l	FLAT l: Flatten a list of lists l to a single list EL y l: *y*^*th*^ element of list l CONS: Adds a new element to the top of the list
Reactants Deletion	⊢ ∀ l x y. **zsyn_delet** l x y = **if** x > y **then** delet (delet l x) y **else** delet (delet l y) x	delet l x: Deletes the element at index x of the list l
Element Deletion	⊢ ∀ l. **delet** l 0 = TL l ∧ ∀ l y. **delet** l (y + 1) = CONS (HD l) ( **delet** (TL l) y)	HD l: Head element of list l TL l: Tail of list l
EVF Matching	⊢ ∀ l e x y. **zsyn_EVF** l e 0 x y = **if** FST (EL 0 e) = HD l **then** (T,zsyn_delet (APPEND (TL l) (SND (EL 0 e))) x y) **else** (F,TL l) ∧ ∀ l e p x y. **zsyn_EVF** l e (p + 1) x y = **if** FST (EL (p + 1) e) = HD l **then** (T,zsyn_delet (APPEND (TL l) (SND (EL (SUC p) e))) x y) **else** **zsyn_EVF** l e p x y	FST: First component of a pair SND: Second component of a pair APPEND: Merges two lists zsyn_delet: Reactants deletion
Recursive Function to model the argument y in function zsyn_EVF	⊢ ∀ l e x. zsyn_recurs1 l e x 0 = zsyn_EVF (zsyn_conjun_intro l x 0) e (LENGTH e - 1) x 0 ∧ ∀ l e x y. zsyn_recurs1 l e x (y + 1) = **if** FST (zsyn_EVF (zsyn_conjun_intro l x (y + 1)) e (LENGTH e - 1) x (y + 1)) ⇔ T **then** zsyn_EVF (zsyn_conjun_intro l x (y + 1)) e (LENGTH e - 1) x (y + 1) **else** zsyn_recurs1 l e x y	LENGTH e: Length of list e zsyn_EVF: EVF Matching zsyn_conjun_intro: Introduction of Z-Conjunction and Z-Interaction
Recursive Function to model the argument x in function zsyn_EVF	⊢ ∀ l e y. **zsyn_recurs2** l e 0 y = **if** FST (zsyn_recurs1 l e 0 y) ⇔ T **then** (T,SND (zsyn_recurs1 l e 0 y)) **else** (F,SND (zsyn_recurs1 l e 0 y)) ∧ ∀ l e x y. **zsyn_recurs2** l e (x + 1) y = **if** FST (zsyn_recurs1 l e (x + 1) y) ⇔ T **then** (T,SND (zsyn_recurs1 l e (x + 1) y)) **else** **zsyn_recurs2** l e x (LENGTH l - 1)	zsyn_recurs1: Recursive function to model the augment y in zsyn_EVF
Final Recursion Function for Zsyntax	⊢ ∀ l e x y. **zsyn_deduct_recurs** l e x y 0 = (T,l) ∧ ∀ l e x y q. **zsyn_deduct_recurs** l e x y (q + 1) = **if** FST (zsyn_recurs2 l e x y) ⇔ T **then** **zsyn_deduct_recurs** (SND (zsyn_recurs2 l e x y)) e (LENGTH (SND (zsyn_recurs2 l e x y))—1) (LENGTH (SND (zsyn_recurs2 l e x y))—1) q **else** (T,SND (zsyn_recurs2 l e (LENGTH l - 1) (LENGTH l- 1)))	zsyn_recurs2: Recursive function to model the augment x in zsyn_EVF
Final Deduction Function for Zsyntax	⊢ ∀ l e. **zsyn_deduct** l e = SND (zsyn_deduct_recurs l e (LENGTH l- 1) (LENGTH l - 1) LENGTH e)	zsyn_deduct_recurs: Recursive Function for calling zsyn_EVF

The Z-Interaction and the introduction of Z-Conjunction (& I) rule jointly enable us to perform a reaction between different molecules during the experiment. This rule is basically the append operation of lists, based on the above data types defined in our formalization. The function zsyn_conjun_intro, given in Table 2, represents this particular rule. It takes a list l and two of its elements x and y, and appends the list of these two elements on its head as shown in Fig 2b.

According to laws of stoichiometry [13], we have to delete the initial reacting molecules from the main list, for which the Z-Conjunction operator is applied. Our formalization of this behavior is represented by the function zsyn_delet, given in Table 2 and depicted in Fig 2c. The function zsyn_delet accepts a list l and two numbers x and y and deletes the *x*^*th*^ and *y*^*th*^ elements of the given list l. The function checks if the index *x* is greater than the index *y*, i.e., *x* > *y*. If the condition is true, then it deletes the *x*^*th*^ element first and then the *y*^*th*^ element. Similarly, if the condition *x* > *y* is false, then it deletes the *y*^*th*^ element first and then the *x*^*th*^ element. In this deletion process, to make sure that the deletion of first element will not affect the index of the other element that has to be deleted, we delete the element present at the higher index of list before the deletion of the lower indexed element.

We aim to build a framework that takes the initial molecules of a biological experiment along with the possible EVFs and enables us to deduce its corresponding final outcomes. Towards this, we first write a function zsyn_EVF, given in Table 2 and depicted in Fig 2d, that takes a list of initial molecules and compares its particular combination with the corresponding EVFs and if a match is found then it adds the newly resulted molecule to initial list after deleting the instance that have already been consumed. The function zsyn_EVF takes a list of molecules l and a list of EVFs e and compares the head element of the list l to all of the elements of the list e. Upon finding no match, this function returns a pair having first element as false (F), which acts as a flag and indicates that there is no match between any of the EVFs and the corresponding molecule, whereas the second element of the pair is the tail of the corresponding list l of the initial molecules. If a match is found, then the function will return a pair with its first element as a true (T), which indicates the confirmation of the match that have been found, and the second element of the pair is the modified list l, whose head is removed, and the second element of the corresponding EVF pair is added at the end of the list and the matched elements are deleted as these have already been consumed.

Next, we have to call the function zsyn_EVF recursively, for the deduction of the final outcome of the experiment and for each of the recursive case, we place each of the possible combinations of the given molecules (elements at indices x and y of list l) at the head of l one by one. This whole process can be done using functions zsyn_recurs1 and zsyn_recurs2, given in Table 2. In the function zsyn_recurs1, we first place the combination of molecules indexed by variables x and y at the top of the list l using the introduction of Z-Conjunction rule. Then, this modified list l is passed to the function zsyn_EVF, which is recursively called by the function zsyn_recurs1. Moreover, we instantiate the variable p of the function zsyn_EVF with the length of the EVF list (LENGTH e - 1) so that every new combination of the list l is compared with all the elements of the list of EVFs e. The function zsyn_recurs1 terminates upon finding a match in the list of EVFs and returns true (T) as the first element of its output pair, which acts as a flag for the status of this match. The second function zsyn_recurs2 checks, if a match in the list of EVFs e is found (if the flag returns true (T)) then it terminates and returns the output list of the function zsyn_recurs1. Otherwise, it recursively checks for the match with all of the remaining values of the variable x. In the case of a match, these two functions zsyn_recurs1 and zsyn_recurs2 have to be called all over again with the new updated list. This iterative process continues until no match is found in the execution of these functions. This overall behaviour can be expressed in HOL Light by the recursive function zsyn_deduct_recurs, given in Table 2. In order to guarantee the correct operation of deduction, we instantiate the variable of recursion (q) with a value that is greater than the total number of EVFs so that the application of none of the EVF is missed. Similarly, in order to ensure that all the combinations of the list l are checked against the entries of the EVF list e, the value LENGTH l - 1 is assigned to both of the variables x and y. Thus, the final deduction function for Zsyntax can be modeled as the function zsyn_deduct, given in Table 2. The function zsyn_deduct accepts the initial list of molecules l and the list of valid EVFs e and returns a list of final outcomes of the experiment under the given conditions. Next, in order to check, if the desired molecule is present in this list (the output of the function zsyn_deduct), we apply the elimination of the Z-Conjunction rule presented as function zsyn_conjun_elimin, given in Table 2. More detail about the behavior of all of these functions can be found in our proof script [63].

These formal definitions enable us to check recursively all of the possible combinations of the molecules, present in the initial list l, against each of the first element of the list of EVFs e. Upon finding a match, the reacting molecules are replaced by their outcome in the initial list of molecules l by applying the corresponding EVF. This process is repeated on the current updated list of molecules until there are no further molecules reacting with each other. The list l at this point contains the post-reaction molecules. Finally, the elimination of the Z-Conjunction rule zsyn_conjun_elimin, given in Table 2, is applied to obtain the desired outcome of the given biological experiment.

In order to prove the correctness of the formal definitions presented above, we verify a couple of key properties of Zsyntax involving operators depicting the vital behaviour of the molecular reactions. The first verified property captures the scenario when there is no reacting molecule present in the initial list of the experiment. As a result of this scenario, the post-experiment molecules are the same as the pre-experiment molecules. The second property deals with the case when there is only one set of reacting molecules in the given initial list of molecules and in this scenario we verify that after the execution of the Zsyntax based experiment, the list of post-experiment molecules contains the products of the reacting molecules minus its reactant along with the remaining non-reacting molecules provided at the beginning of the experiment. We formally specified both of these properties, representing the no reaction and single reaction scenarios in higher-order logic using the formal definitions presented earlier in this section. The formal verification results about these properties are given in Table 3 and more details can be found in the description of their formalization [18, 63]. The formalization presented in this section provides an automated reasoning support for the Zsyntax based molecular biological experiments within the sound core of HOL Light theorem prover.

**Table 3:** Formal verification of Zsyntax properties.

Name	Formalized Form	Description
**Case:1** No Reaction	⊢ ∀ e l. **A1:** ∼(NULL e) ∧ **A2:** ∼(NULL l) ∧ **A3:** (∀ a x y. MEM a e ∧ x < LENGTH l ∧ y < LENGTH l ⇒ ∼MEM (FST a) [HD (zsyn_conjun_intro l x y)]) ⇒ zsyn_deduct l e = l	e: List of EVFs l: List of molecules **A1**: List e is non-empty **A2**: List l is non-empty **A3**: The formalization of the no-reaction-possibility condition **Conclusion**: Both the pre and post-experiment lists of molecules are the same
**Case:2** Single Reaction	⊢ ∀ e l z x’ y’. **A1:** ∼(NULL e) ∧ **A2:** ∼(NULL (SND (EL z e))) ∧ **A3:** 1 < LENGTH l ∧ **A4:** x’ ≠ y’ ∧ **A5:** x’ < LENGTH l ∧ **A6:** y’ < LENGTH l ∧ **A7:** z < LENGTH e ∧ **A8:** ALL_DISTINCT (APPEND l (SND (EL z e))) ∧ **A9:** (∀ a b. a ≠ b ⇒ FST (EL a e) ≠ FST (EL b e)) ∧ **A10:** (∀ k x y. x < LENGTH k ∧ y < LENGTH k ∧ (∀ j. MEM j k ⇒ MEM j l ∨ (∃ q. MEM q e ∧ MEM j (SND q)) ⇒ **if** (EL x k = EL x’ l) ∧ (EL y k = EL y’ l) **then** HD (zsyn_conjun_intro k x y) = FST (EL z e) **else** ∀ a. MEM a e ⇒ FST a ≠ HD (zsyn_conjun_intro k x y)) ⇒ zsyn_deduct l e = zsyn_delet (APPEND l (SND (EL z e))) x’ y’	e: List of EVFs l: List of molecules **A1-A2**: The list e and the second element of the pair at index z of the list e is non-empty **A3**: List l, i.e., the list of initial molecules, contains at least two elements **A4**: The indices x’ and y’ are distinct **A5-A7**: The indices x’, y’ and z fall within the range of elements of their respective lists of molecules l or EVFs e **A8**: All elements of the list l and the resulting molecules of the EVF at index z are distinct **A9**: All first elements of the pairs in list e are distinct **A10**: It models the scenario where there is only one pair of reactants present in the reaction **Conclusion**: The scenario when the resulting element, available at the location z of the EVF list, is appended to the list of molecules while the elements available at the indices x’ and y’ of l are removed during the execution of the function zsyn_deduct on the given lists l and e

### Formalization of reaction kinetics

Reaction kinetics [64] is the study of rates at which biological processes interact with each other and how the corresponding processes are affected by these reactions. The rate of a reaction provides the information about the evolution of the concentration of the species (e.g., molecules) over time. A process is basically a chain of reactions, called pathway, and the investigation about the rate of a process implies the rate of these pathways. Generally, biological reactions can be either irreversible (unidirectional) or reversible (bidirectional). We formally define this fact by an inductive enumerating data-type reaction_type, given in Table 4.

**Table 4:** Definitions of reaction kinetics formalization.

Name	Formalized Form	Description
**Biological Reaction**		
Reaction Type	define_type “**reaction_type**” = irreversible | reversible”	Reaction type (reversible or irreversible) defined by an inductive enumerating data-type
Biological Reaction	new_type_abbrev “**bio_reaction**”, :(reaction_type × ((N × R)list × (N × R)list × (R × R)))	Biological reaction is a pair with reaction type as the first element and a 3-tuple as the second element with the following components: (N × R)list: List of reactants, where N is the stiochiometry and R represents the concentration of a reactant (N × R)list: List of products, where N is the stiochiometry and R represents the concentration of a product (R × R): The first element R is the forward kinetic rate constant and the second element R is the reverse kinetic rate constant for reversible reaction. A zero here indicates a irreversible reaction.
**Flux Vector**		
Product of the Concentrations	⊢ ∀ h t. **flux_irr** [ ] = &1 ∧ **flux_irr** (CONS h t) = **if** FST h = 0 **then** **flux_irr** t **else** SND h pow FST h ∗ **flux_irr** t	It takes a list of reactants in the form of a pair and returns a real number, which is the product of the concentration raised to the power of the stoichiometry of all the reactants in the reaction.
Flux of an Irreversible Reaction	⊢ ∀ products_list rate reactants_list. **gen_flux_irreversible** reactants_list products_list rate = rate ∗ flux_irr reactants_list	It takes a list of reactants, a list of products and the first element of the kinetic rate constant pair and returns the flux of an irreversible reaction.
Flux of a Reversible Reaction	⊢ ∀ rate_1 reactants_list rate_2 products_list. **gen_flux_reversible** reactants_list products_list rate_1 rate_2 = rate_1 ∗ flux_irr reactants_list - rate_2 ∗ flux_irr products_list	It takes a list of reactants, a list of products and the forward kinetic rate constant, reverse kinetic rate constant and returns the flux of a reversible reaction.
Flux of a Single Reaction	⊢ ∀ t R P k1 k2. **flux_sing** (t,R,P,k1,k2) = **if** t = irreversible **then** gen_flux_irreversible R P k1 **else** gen_flux_reversible R P k1 k2	The defintions gen_flux_irreversible and gen_flux_reversible are combined into a uniform defintion. The function flux_sing takes a biological reaction, which can be a reversible or irreversible reaction, and returns the corresponding flux of that reaction.
Flux Vector	⊢ ∀ M. **flux** M = vector (MAP flux_sing M)	It takes a list of biological reactions and returns flux vector **v**.
**Stoichiometric Matrix**		
Column of the Stoichiometric Matrix	⊢ ∀ h t h2 h1 t1 t2. **stioch_mat_column** [ ] [ ] = [ ] ∧ **stioch_mat_column** (CONS h t) [ ] = [ ] ∧ **stioch_mat_column** [ ] (CONS h t) = [ ] ∧ **stioch_mat_column** (CONS h1 t1) (CONS h2 t2) = CONS (&(FST h2)—&(FST h1)) ( **stioch_mat_column** t1 t2)	It accepts a list of the reactants and a list of products and returns a list containing the corresponding column of the stoichiometric matrix.
Vector of the Stoichiometric Matrix Column	⊢ ∀ t k1 k2 R P. **st_matrix_sing** (t,R,P,k1,k2) = vector (stioch_mat_column R P)	It takes a single biological reaction (*bio*_*reaction*) and returns a vector ($R^{m}$), which corresponds to the column of the stoichiometric matrix.
Stoichiometric Matrix	⊢ ∀ M. **st_matrix** M = vector (MAP st_matrix_sing M)	It takes a list of biological reactions and returns a stiochiometric matrix (in transposed form) using the MAP function, which applies the function st_matrix_sing on every element of the list *M*.
**Vector of Derivative**		
Derivative of a List of Functions	⊢ ∀ h t x. **map_real_deriv** [ ] x = [ ] ∧ **map_real_deriv** (CONS h t) x = APPEND [real_derivative h x] ( **map_real_deriv** t x)	It takes a list containing the concentrations of all the species taking part in the reaction and maps a real derivative over each function of the list using the function real_derivative, which represents the real-valued derivative of a function
Derivative of a Vector	⊢ ∀ L t. **entities_deriv_vec** L t = vector (map_real_deriv L t)	It accepts a list containing the concentrations of species and returns a vector with each element represented in the form of a real-valued derivative, which is left-hand side of vector equation, i.e., $\frac{d[X]}{dt}$.

In order to analyze a biological process, we need to know its kinetic reaction based model, which comprises of a set of *m* species, *X* = {*X*_1_, *X*_2_, *X*_3_,…, *X*_*m*_} and a set of *n* reactions, *R* = {*R*_1_, *R*_2_, *R*_3_,…, *R*_*n*_}. An irreversible reaction *R*_*j*_, {1 ≤ *j* ≤ *n*} can generally be written as: $R_{j}:s_{1,j}X_{1}+s_{2,j}X_{2}+\ldots +s_{m,j}X_{m}\overset{k_{j}}{\to }{\overset{´}{s}}_{1,j}X_{1}+{\overset{´}{s}}_{2,j}X_{2}+\ldots +{\overset{´}{s}}_{m,j}X_{m}$. Similarly, a reversible reaction *R*_*j*_, {1 ≤ *j* ≤ *n*} can be described as: $R_{j}:s_{1,j}X_{1}+s_{2,j}X_{2}+\ldots +s_{m,j}X_{m}\overset{{k_{j}}^{f}}{\underset{{k_{j}}^{r}}{⇄}}{\overset{´}{s}}_{1,j}X_{1}+{\overset{´}{s}}_{2,j}X_{2}+\ldots +{\overset{´}{s}}_{m,j}X_{m}$. The coefficients $s_{1,j},s_{2,j},\ldots ,s_{m,j},{\overset{´}{s}}_{1,j},{\overset{´}{s}}_{2,j},\ldots ,{\overset{´}{s}}_{m,j}$ are the non-negative integers and represent the stoichiometries of the species taking part in the reaction. The non-negative integer *k*_*j*_ is the kinetic rate constant of the irreversible reaction. The non-negative integers ${k_{j}}^{f}$ and ${k_{j}}^{r}$ are the forward and reverse kinetic rate constants of the reversible reaction, respectively [65]. In a biological reaction, we model a biological entity as a pair (N, R), where the first element represents the stoichiometry and the second element is the concentration of the molecule. We formally model a biological reaction as the type abbreviation bio_reaction [63], given in Table 4.

The dynamic behavior of the biological systems is described by a set of ordinary differential equations (ODEs) and the evolution of the system is captured by analyzing the change in the concentration of the species (i.e., time derivatives): $\frac{d[X_{i}]}{dt}=\sum_{j=1}^{n}n_{i,j}v_{j}$, where *n*_*i*, *j*_ is the stoichiometric coefficient of the molecular species *X*_*i*_ in reaction *R*_*j*_ (i.e., $n_{i,j}={\overset{´}{s}}_{i,j}-s_{i,j}$). The parameter *v*_*j*_ represents the flux of the reaction *R*_*j*_, which can be computed by the law of mass action [14], i.e., the rate (also called flux) of a reaction is proportional to the concentration of the reactant (c) raised to the power of its stoichiometry (s), i.e., *c*^*s*^. We define the function gen_flux_irreversible, given in Table 4, to obtain the flux of an irreversible reaction [63].

A reversible reaction can be divided into two irreversible reactions with the forward kinetic rate constant and the reverse kinetic rate constant, respectively. The rate/flux of a reversible reaction is obtained by taking the differences of the fluxes of the two irreversible reactions. We formally define the flux of a reversible reaction by the function gen_flux_reversible, given in Table 4. Next, we combine the functions gen_flux_irreversible and gen_flux_reversible into one uniform function flux_single (Table 4)[63] to obtain the flux of a single reaction.

For all reactions from 1 to *n* of a biological system, the flux becomes a flux vector as **v** = (*v*_1_, *v*_2_,…, *v*_*n*_)^*T*^ and the system of ODEs can be written in the vectorial form as: $\frac{d[X]}{dt}=Nv$, where [**X**] = (*X*_1_ *X*_2_,…, *X*_*n*_)^*T*^ is a vector of the concentration of all of the species participating in the reaction and *N* is the stoichiometric matrix of order *m* × *n*. We can obtain the flux vector **v** for a chain of reactions of a biological system by the function flux [63], given in Table 4.

Next, we formalize the notion of stoichiometric matrix *N* by the function st_matrix [63] given in Table 4. Finally, in order to formalize the left-hand side of above vector equation, i.e., $\frac{d[X]}{dt}$, we define a function entities_deriv_vec which takes a list containing the concentrations of all species and returns a vector with each element represented in the form of a real-valued derivative.

We can utilize this infrastructure to model arbitrary biological networks consisting of any number of reactions. For example, a biological network consisting of a list of E biological species and M biological reactions can be formally represented by the following general kinetic model:

$$\begin{array}{c} \left(\left(\text{entities}\_\text{deriv}\_\text{vec}\;E\;t\right):\text{real}\hat{\;}m\right)=\text{transp}\left(\left(\text{st}\_\text{matrix}\;M\right):\text{real}\hat{\;}m\hat{\;}n\right)\;**\;\text{flux}\;M \end{array}$$

We used the formalization of the reaction kinetics to verify some generic properties of biological reactions, such as irreversible consecutive reactions, reversible and irreversible mixed reactions. The main idea is to express the given biological network as a kinetic model and verify that the given solution (mathematical expression) of each biological entity satisfies the resulting set of coupled differential equations. This verification is quite important as such expressions are used to predict the outcomes of various drugs and to understand the time evolution of different molecules in the reactions of the biological systems.

#### The irreversible consecutive reactions

**Fig 3:**
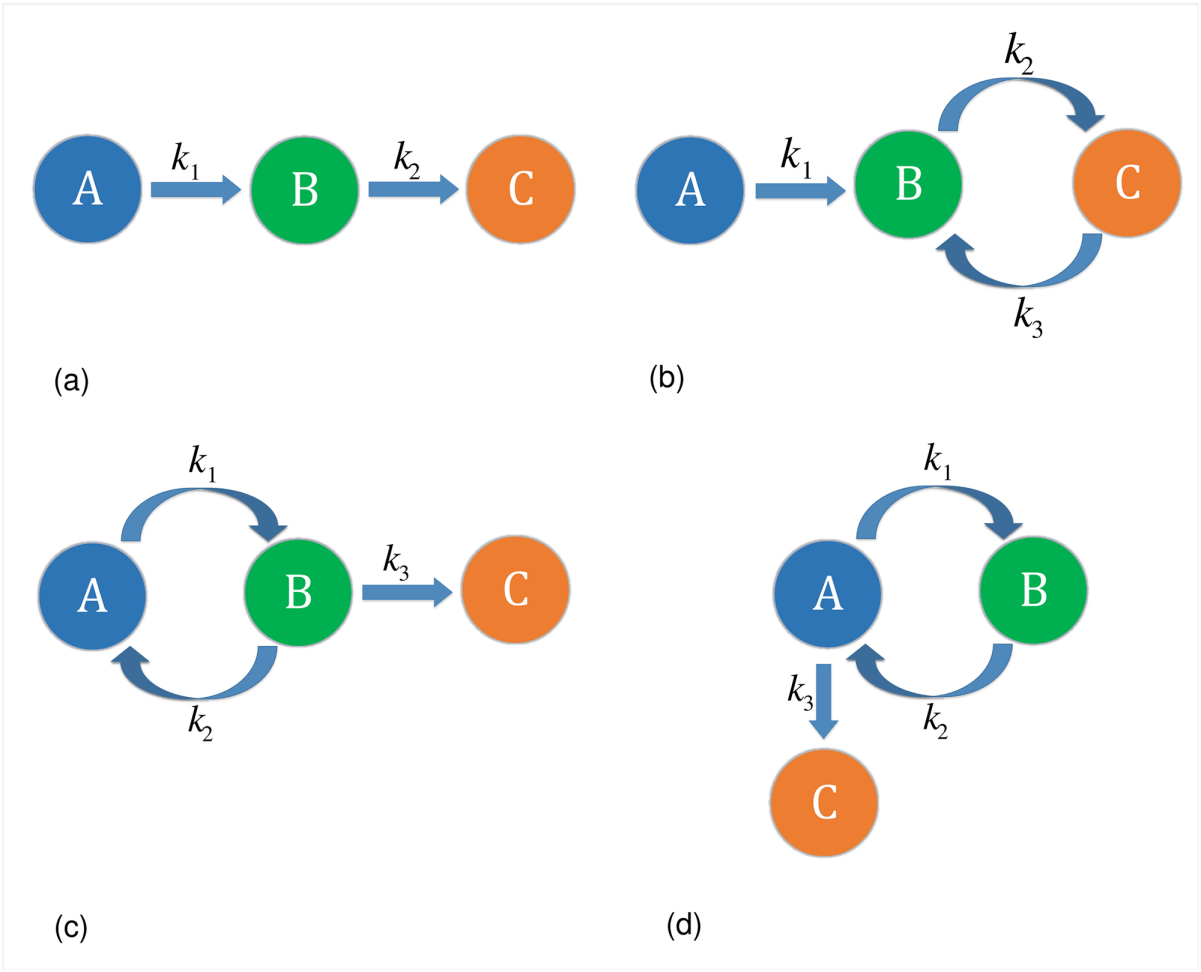
Reaction schemes. (a) Irreversible Consecutive Reactions (b) Consecutive Reactions with the Second Step being Reversible (c) Consecutive Reactions with the First Step as a Reversible Reaction (d) Consecutive Reactions with a Reversible Step.

We consider a general irreversible consecutive reaction scheme as shown in Fig 3a. In the first reaction, A is the reactant and B is the product whereas *k*_1_ represents the kinetic rate constant of the reaction. Similarly, in the second reaction, B is the reactant, C is the product and *k*_2_ is its kinetic rate constant. We formally model this reaction scheme as a HOL Light function rea_sch_01, given in Table 5, and the formalization details are available as a technical report [66]. We moreover verify the solution of its kinetic model in HOL Light. The formal verification results are given in Table 6 [63].

**Table 5:** Formal models of generic reaction schemes.

Name	Formalized Form	Description
The Irreversible Consecutive Reactions	⊢ ∀ k1 A B C t k2. rea_sch_01 A B C k1 k2 t = [irreversible,[1,A t; 0,B t; 0,C t],[0,A t; 1,B t; 0,C t],k1,&0; irreversible,[0,A t; 1,B t; 0,C t],[0,A t; 0,B t; 1,C t],k2,&0]	rea_sch_01: It accepts the concentrations of the species A, B, C, the kinetic rate constants k1, k2, a real-valued time variable t and returns a list of two irreversible biological reactions (*bio*_*reaction*).
The Consecutive Reactions with the Second Step Being Reversible	⊢ ∀ k1 A B C t k2 k3. rea_sch_02 A B C k1 k2 k3 t = [irreversible,[1,A t; 0,B t; 0,C t],[0,A t; 1,B t; 0,C t],k1,&0; reversible,[0,A t; 1,B t; 0,C t],[0,A t; 0,B t; 1,C t],k2,k3]	rea_sch_02: It accepts the concentrations of the species A, B, C, the kinetic rate constants k1, k2, k3, a real-valued time variable t and returns the list of biological reactions (*bio*_*reaction*).
The Consecutive Reactions with First Step as a Reversible Reaction	⊢ ∀ k1 k2 A B C t k3. rea_sch_03 A B C k1 k2 k3 t = [reversible,[1,A t; 0,B t; 0,C t],[0,A t; 1,B t; 0,C t],k1,k2; irreversible,[0,A t; 1,B t; 0,C t],[0,A t; 0,B t; 1,C t],k3,&0]	rea_sch_03: It takes the concentrations of the species A, B, C, the kinetic rate constants k1, k2, k3 and the time variable t and returns the corresponding list of biological reactions (*bio*_*reaction*).
The Consecutive Reactions with a Reversible Step	⊢ ∀ k1 k2 A B C t k3. rea_sch_04 A B C k1 k2 k3 t = [reversible,[1,A t; 0,B t; 0,C t],[0,A t; 1,B t; 0,C t],k1,k2; irreversible,[1,A t; 0,B t; 0,C t],[0,A t; 0,B t; 1,C t],k3,&0]	rea_sch_04: It accepts the concentrations of the species A, B, C, the kinetic rate constants k1, k2, k3, the time variable t and returns the list of corresponding biological reactions (*bio*_*reaction*).

**Table 6:** Formal verification of reaction kinetics properties.

Name	Formalized Form	Description
The Irreversible Consecutive Reactions	⊢ ∀ A B C t k_1_ k_2_. **A1:** 0 < k_1_ ∧ **A2:** 0 < k_2_ ∧ **A3:** (k_2_ −k_1_ ≠0) ∧ **A4:** A(0) = A_0_ ∧ **A5:** B(0) = 0 ∧ **A6:** C(0) = 0 ∧ **A7:** ∀t. A(t) = A_0_ e^(−k_1_t)^ ∧ **A8**: $\forall t.\;B\left(t\right)=A_{0}\frac{k_{1}}{\left(k_{2}-k_{1}\right)}\left(e^{-k_{1}t}-e^{-k_{2}t}\right)\;\wedge$ **A9**: $\forall t.\;C\left(t\right)=A_{0}(1-\frac{k_{2}}{\left(k_{2}-k_{1}\right)}e^{-k_{1}t}-\frac{k_{1}}{\left(k_{1}-k_{2}\right)}e^{-k_{2}t})$ ⇒ entities_deriv_vec [A; B; C] t = transp (st_matrix (rea_sch_01 A B C k_1_ k_2_ t)) ∗ ∗ flux (rea_sch_01 A B C k_1_ k_2_ t)	∗ ∗ : Matrix-vector multiplication rea_sch_01: Formal model of the given reaction scheme transp: Transpose of a matrix **A1-A2**: The kinetic rate constants of all the reactions are non-negative. **A3**: The denominators of the expressions of B t and C t are not zero in order to avoid singularities **A4-A6**: These are the initial concentrations of the species **A7-A9**: The concentrations of the species A, B and C at any time t (solutions of the ODE model) **Conclusion**: It describes the ODE model (Vector Equation) for the given reaction scheme
The Consecutive Reactions with the Second Step being Reversible	⊢ ∀ A B C t k_1_ k_2_ k_3_. **A1:** 0 < k_1_ ∧ **A2:** 0 < k_2_ ∧ **A3:** 0 < k_3_ ∧ **A4:** A(0) = A_0_ ∧ **A5:** B(0) = 0 ∧ **A6:** C(0) = 0 ∧ **A7:** r_1_ = k_1_ ∧ **A8:** r_2_ = k_2_ +k_3_ ∧ **A9:** r_1_ ≠r_2_ ∧ **A10:** ∀t. A(t) = A_0_ e^(−k_1_t)^ ∧ **A11**: $\forall t.\;B\left(t\right)=k_{1}A_{0}(\frac{k_{3}}{r_{1}r_{2}}+\frac{r_{2}-k_{3}}{r_{1}\left(r_{1}-r_{2}\right)}e^{-r_{2}t}+\frac{k_{3}-r_{1}}{r_{1}\left(r_{1}-r_{2}\right)}e^{-r_{1}t})\;\wedge$ **A12**: $\forall t.\;C\left(t\right)=k_{1}k_{2}A_{0}(\frac{1}{r_{1}r_{2}}+\frac{1}{r_{1}\left(r_{1}-r_{2}\right)}e^{-r_{1}t}+\frac{1}{r_{2}\left(r_{1}-r_{2}\right)}e^{-r_{2}t})\;\wedge$ ⇒ entities_deriv_vec [A; B; C] t = transp (st_matrix (rea_sch_02 A B C k_1_ k_2_ k_3_ t)) ∗ ∗ flux (rea_sch_02 A B C k_1_ k_2_ k_3_ t)	rea_sch_02: Formal model of the given reaction scheme **A1-A3**: The kinetic rate constants of all the reactions are non-negative. **A4-A6**: These are the initial concentrations of the species **A7-A8**: These are introduced to simplify the expressions for the concentrations of the species **A9**: It, along with the first three assumptions (A1-A3), ensures that the denominators of the expressions for B t and C t are not zero in order to avoid singularities **A10-A12**: The concentrations of the species A, B and C at any time t (solutions of the ODE model) **Conclusion**: It describes the ODE model for the given reaction scheme
The Consecutive Reactions with the First Step being Reversible	⊢ ∀ A B C t k_1_ k_2_ k_3_. **A1:** 0 < k_1_ ∧ **A2:** 0 < k_2_ ∧ **A3:** 0 < k_3_ ∧ **A4:** A(0) = A_0_ ∧ **A5:** B(0) = 0 ∧ **A6:** C(0) = 0 ∧ **A7:** r_1_ r_2_ = k_1_ k_3_ ∧ **A8:** r_1_ +r_2_ = k_1_ +k_2_ +k_3_ ∧ **A9:** r_1_ ≠0 ∧ **A10:** r_2_ ≠0 ∧ **A11:** r_1_ ≠r_2_ ∧ **A12**: $\forall t.\;A\left(t\right)=\frac{A_{0}}{r_{2}-r_{1}}(\left(k_{2}+k_{3}-r_{1}\right)e^{\left(-r_{1}t\right)}-\left(k_{2}+k_{3}-r_{2}\right)e^{\left(-r_{2}t\right)})\;\wedge$ **A13**: $\forall t.\;B\left(t\right)=\frac{A_{0}k_{1}}{r_{2}-r_{1}}(e^{-r_{1}t}-e^{-r_{2}t})\;\wedge$ **A14**: $\forall t.\;C\left(t\right)=A_{0}(1+\frac{k_{1}k_{3}}{r_{1}\left(r_{1}-r_{2}\right)}e^{-r_{1}t}+\frac{k_{1}k_{3}}{r_{2}\left(r_{2}-r_{1}\right)}e^{-r_{2}t})\;\wedge$ ⇒ entities_deriv_vec [A; B; C] t = transp (st_matrix (rea_sch_03 A B C k_1_ k_2_ k_3_ t)) ∗ ∗ flux (rea_sch_03 A B C k_1_ k_2_ k_3_ t)	rea_sch_03: Formal model of the given reaction scheme **A1-A3**: The kinetic rate constants of all the reactions are non-negative. **A4-A6**: These are the initial concentrations of the species **A7-A8**: These are introduced to simplify the expressions for the concentrations of the species **A9-A11**: The denominators of the expressions of A t, B t and C t are not zero in order to avoid singularities **A12-A14**: The concentrations of the species A, B and C at any time t (solutions of the ODE model) **Conclusion**: It describes the ODE model for the given reaction scheme
The Consecutive Reactions with a Reversible Step	⊢ ∀ A B C t k_1_ k_2_ k_3_. **A1:** 0 < k_1_ ∧ **A2:** 0 < k_2_ ∧ **A3:** 0 < k_3_ ∧ **A4:** A(0) = A_0_ ∧ **A5:** B(0) = 0 ∧ **A6:** C(0) = 0 ∧ **A7:** r_1_ r_2_ = k_2_ k_3_ ∧ **A8:** r_1_ +r_2_ = k_1_ +k_2_ +k_3_ ∧ **A9:** r_1_ ≠0 ∧ **A10:** r_2_ ≠0 ∧ **A11:** r_1_ ≠r_2_ ∧ **A12**: $\forall t.\;A\left(t\right)=A_{0}(\frac{k_{2}-r_{1}}{r_{2}-r_{1}}e^{-r_{1}t}-\frac{k_{2}-r_{2}}{r_{2}-r_{1}}e^{-r_{2}t})\;\wedge$ **A13**: $\forall t.\;B\left(t\right)=\frac{k_{1}A_{0}}{r_{2}-r_{1}}(e^{-r_{1}t}-e^{-r_{2}t})\;\wedge$ **A14**: $\forall t.\;C\left(t\right)=A_{0}(1+\frac{k_{3}\left(k_{2}-r_{1}\right)}{r_{1}\left(r_{1}-r_{2}\right)}e^{-r_{1}t}+\frac{k_{3}\left(k_{2}-r_{2}\right)}{r_{2}\left(r_{2}-r_{1}\right)}e^{-r_{2}t})\;\wedge$ ⇒ entities_deriv_vec [A; B; C] t = transp (st_matrix (rea_sch_04 A B C k_1_ k_2_ k_3_ t)) ∗ ∗ flux (rea_sch_04 A B C k_1_ k_2_ k_3_ t)	rea_sch_04: Formal model of the given reaction scheme **A1-A3**: The kinetic rate constants of all the reactions are non-negative. **A4-A6**: These are the initial concentrations of the species **A7-A8**: These are introduced to simplify the expressions for the concentrations of the species **A9-A11**: The denominators of the expressions of A t, B t and C t are not zero in order to avoid singularities **A12-A14**: The concentrations of the species A, B and C at any time t (solutions of the ODE model) **Conclusion**: It describes the ODE model for the given reaction scheme

#### The consecutive reactions with the second step being reversible

The second reaction scheme consists of the consecutive reactions with the second step being reversible as shown in Fig 3b. In the irreversible reaction, A and B are the reactant and product, respectively, whereas *k*_1_ is the kinetic rate constant of the reaction. Since any reversible reaction can be written as two irreversible reactions, so the first irreversible reaction has B, C and the forward kinetic reaction constant *k*_2_ as the reactant, product and the kinetic rate constant, respectively. Similarly, the parameters C, B and *k*_3_ are the reactant, product and kinetic rate constant of the second irreversible reaction, respectively. We formally model this scheme as a HOL Light function rea_sch_02 [66] (Table 5) and then verified the solution for its ODE model given in Table 6 [63].

#### The consecutive reactions with the first step as a reversible reaction

In this scheme, the first reaction is reversible and the second reaction is irreversible as shown in Fig 3c. The reversible reaction can be equivalently written as two irreversible reactions with *k*_1_ and *k*_2_ as their kinetic rate constants. In the first irreversible reaction, A and B are the reactant and product, respectively, whereas in the second reaction, B and A are the reactant and product, respectively. For the second step, B, C and *k*_3_ are the reactant, product and kinetic rate constant, respectively. The verified solution of the ODE model corresponding to this reaction scheme (rea_sch_03 [66], given in Table 5) is given in Table 6.

#### The consecutive reactions with a reversible step

In this reaction scheme, we consider the consecutive reactions with one reversible and one irreversible reaction step as shown in Fig 3d. The ODE model and solution corresponding to this reaction scheme (rea_sch_04 [66], given in Table 5) are given in Table 6.

This completes our formal verification of some commonly used reaction schemes. The verification of these solutions requires user interaction but the strength of these theorems lies in the fact that they have been verified for arbitrary values of parameters, such as *k*_1_ and *k*_2_, etc. This is a unique feature of higher-order-logic theorem proving that is not possible in the case of simulation where such continuous expressions are tested for few samples of such parameters. Another important aspect is the explicit presence of all assumptions required to verify the set of ODEs. For example, such assumptions for the above-mentioned reaction schemes are not mentioned in *Korobov et al.*’s paper [67]. More details about the formalization of all above-mentioned types and functions and the formal verification of all above properties, and its source code can be found on our project’s webpage [63].

### Case studies

In this section, we use our proposed framework to formally reason about three case studies: In the first, we formally analyse the reaction involving the phosphorylation of TP53 using our formalization of Zsyntax. In the second, we formally derive the time evolution expressions of different tumor cell types, which are used to predict the tumor population and volume at a given time instant, using our formalization of reaction kinetics. In the third, we take another model for the growth of tumor cells and perform both the Zsyntax and reaction kinetic based formal analysis using our proposed formalizations presented in the *Result* section of the paper.

#### TP53 phosphorylation

**Fig 4:**
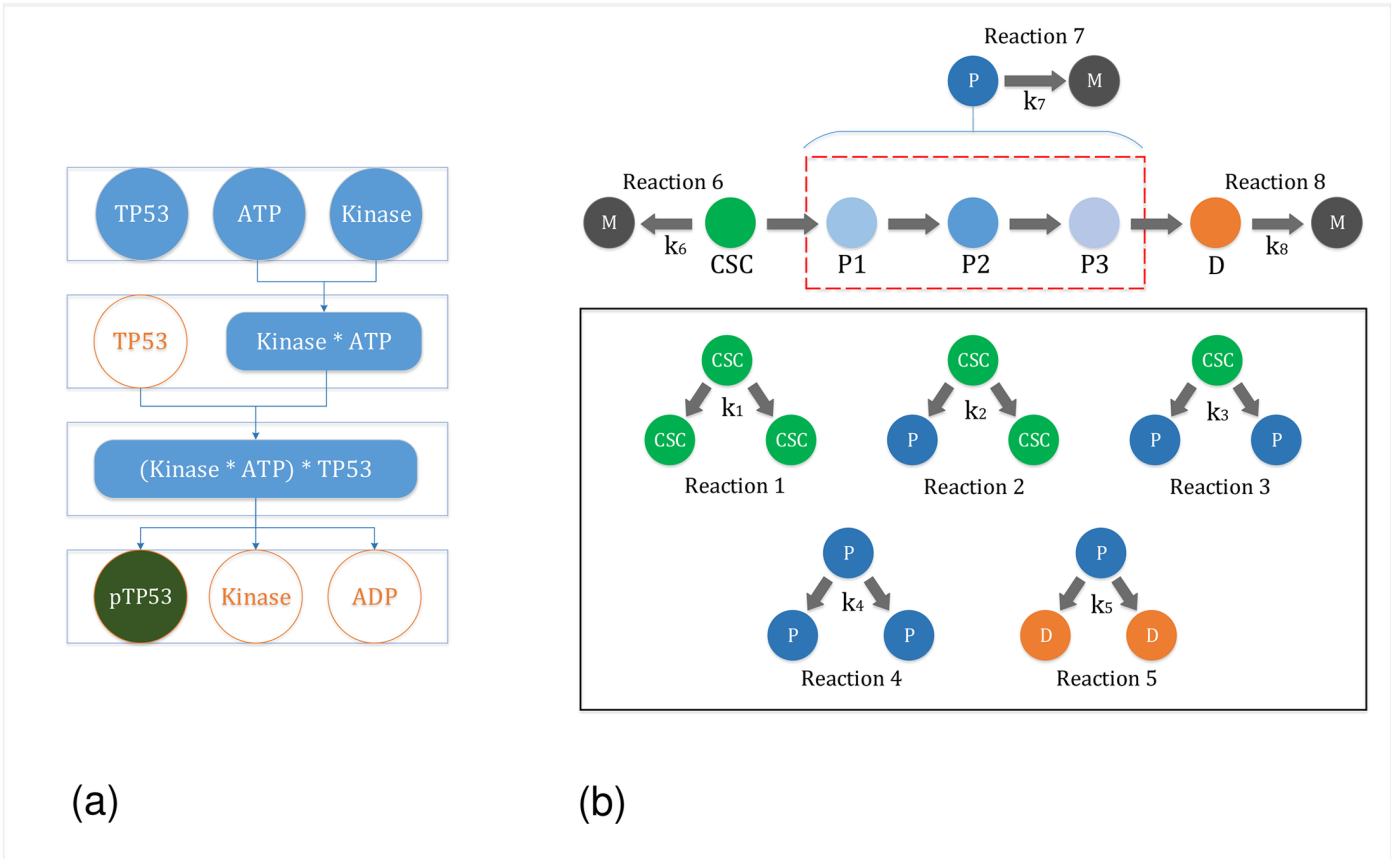
Case studies. (a) Reaction Representing the TP53 Phosphorylation (b) Model for the Tumor Growth [20].

TP53 gene encodes p53 protein, which plays a crucial role in regulating the cell cycle of multicellular organisms and works as a tumour suppressor for preventing cancer [13]. The pathway leading to TP53 phosphorylation (p(TP53)) is shown in Fig 4a. The green-colored circle represents the desired product, whereas, the blued-colored circles describe the chemical interactions in the pathway. Similarly, each rectangle in Fig 4a contains the total number of molecules at a given time. It can be clearly seen from the figure that whenever a biological reaction results into a product, the reactants get consumed, which satisfies the stoichiometry of a reaction. Now, we present the formal verification of pathway deduction from TP53 to p(TP53) using our formalization of Zsyntax, presented in the last section.

In classical Zsyntax format, the reaction of the pathway leading from TP53 to p(TP53) [13] can be represented by a theorem as TP53 & ATP & Kinase ⊢ p(TP53). Based on our formalization, it can be defined as follows:

**Theorem 1**. The reaction of the pathway leading from TP53 to p(TP53)

⊢ DISTINCT [TP53; ATP; Kinase; ADP; pTP53] ⇒

 zsyn_conjun_elimin (zsyn_deduct [[TP53];[ATP];[Kinase]]

  [([Kinase;ATP],[[ATP;Kinase]]);

   ([ATP;Kinase;TP53],[[Kinase];[pTP53];[ADP]])]) [pTP53] = [[pTP53]]

In the above theorem, the first argument of the function zsyn_deduct represents the list of initial aggregate (IA) of molecules that are present at the start of the reaction, whereas the second argument is the list of valid EVFs for this reaction specified in the form of pairs and include the molecules (ATP, Kinase, etc.). These are obtained from wet lab experiments, as reported by *Boniolo et al.* [13]. We use the HOL Light function DISTINCT to ensure that all molecule variables (from IA and EVFs) used in this theorem represent distinct molecules. Thus, the final list of molecules is deduced under these particular conditions using the function zsyn_deduct. Finally, if the molecule pTP53 is present in the post-reaction list of molecules, it will be obtained after the application of the function zsyn_conjun_elimin, as previously described. Additionally, in order to automate the verification process, we developed a simplifier Z_SYNTAX_SIMP [63], which is based on some derived rules and already available HOL Light tactics that simplified the manual reasoning and thus allowed us to formally verify Theorem 1 automatically. It is important to note that formalization of Zsyntax was quite a tedious effort but it took only 6 lines of code for the verification of the theorem of pathway deduction from TP53 to pTP53 in HOL Light, which clearly illustrates the effectiveness of our foundational work.

We have shown that our formalization is capable of modeling molecular reactions using Zsyntax inference rules, i.e., given an IA **A** and a set of possible EVFs, our proposed framework can derive a final aggregate (FA) **B** from **A** automatically. If it fails to deduce **B**, our formalism still provides all the intermediate steps to the biologist so that he can figure out the possible causes of failures, by carefully examining the intermediate steps of the reaction.

#### Formal analysis of tumor growth based on Cancer Stem Cells (CSC)

According to the Cancer Stem Cell (CSC) hypothesis [68], malignant tumors (cancers) are originally initiated by different tumor cells, which have similar physiological characterises as of normal stem cells in the human body. This hypothesis explains that the cancer cell exhibits the ability to self-renew and can also produce different types of differentiated cells. The mathematical and computational modeling of cancers can provide an in-depth understanding and the prediction of required parameters to shape the clinical research and experiments. This can result in efficient planning and therapeutic strategies for accurate patient prognosis. In this paper, we consider a kinetic model of cancer based on the cancer stem cell (CSC) hypothesis, which was recently proposed in *Molina-Pena et al.*’s paper [20]. In this model, four types of events are considered: 1) CSC self-renewal; 2) maturation of CSCs into P cells; 3) differentiation to D cells; and 4) death of all cell subtypes. All of these types of reactions are driven by different rate constants as shown in Fig 4b.

In the following, we provide the possible reactions in the considered model of cancer [20]:

1. Expansion of CSCs can be accomplished through symmetric division, where one CSC can produce two CSCs, i.e., $\text{CSC}\overset{{\text{k}}_{1}}{\to }2\text{CSC}$.
2. A CSC can undergo asymmetric division (whereby one CSC gives rise to another CSC and a more differentiated progenitor (P) cell). This P cell possesses intermediate properties between CSCs and differentiated (D) cells, i.e., $\text{CSC}\overset{{\text{k}}_{2}}{\to }\text{CSC}+\text{P}$.
3. The CSCs can also differentiate to P cells by symmetric division, i.e., $\text{CSC}\overset{{\text{k}}_{3}}{\to }2\text{P}$.
4. The P cells can either self-renew, with a decreased capacity compared to CSCs, or they can differentiate to D cells, i.e., $\text{P}\overset{{\text{k}}_{4}}{\to }2\text{P}$, $\text{P}\overset{{\text{k}}_{5}}{\to }2\text{D}$.
5. All cellular subtypes can undergo cell death (M), i.e., $\text{CSC}\overset{{\text{k}}_{6}}{\to }\text{M}$, $\text{P}\overset{{\text{k}}_{7}}{\to }\text{M}$, $\text{D}\overset{{\text{k}}_{8}}{\to }\text{M}$.

In order to reduce the complexity of the resulting model, only three subtypes of cells are considered: CSCs, transit amplifying progenitor cells (P), and terminally differentiated cells (D) as shown in Fig 4b. This assumption is consistent with several experimental reports [20]. Our main objective is to derive the mathematical expressions, which characterize the time evolution of CSC, P and D. Concretely, the values of these cells should satisfy the set of differential equations that arise in the kinetic model of the proposed tumor growth. Once the expressions of all cell types are known, the total number of tumor cells (*N*) in the human body can be computed by the formula *N*(*t*) = *CSC*(*t*) + *P*(*t*) + *D*(*t*). Furthermore, the tumor volume (*V*) can be calculated by the formula *V*(*t*) = 4.18 × 10^6^ *N*(*t*), considering that the effective volume contribution of a spherically shaped cell in a spherical tumor (i.e., 4.18 × 10^−6^ *mm*^3^/*cell*).

We formally model the tumor growth model and verify the time evolution expressions for CSC, P and D that satisfy the general kinetic model. We formally represent this requirement in the following important theorem:

**Theorem 2**. Time Evolution Verification of Tumor Growth Model

⊢ ∀ k_1_ k_2_ k_3_ k_4_ k_5_ k_6_ k_7_ CSC P D M t k_8_.

 **A1:** ((−k_1_ +k_3_ +k_4_ −k_5_ +k_6_ −k_7_ )(−k_1_ +k_3_ +k_6_ −k_8_ )(−k_4_ +k_5_ +k_7_ −k_8_ )≠0) ∧

 **A2:** (k_1_ −k_3_ −k_4_ +k_5_ −k_6_ +k_7_ ≠0) ∧

 **A3:** ∀t. CSC(t) = e^(k_1_−k_3_−k_6_)^ ∧

 **A4**: $\;\forall t.\;P\left(t\right)=\frac{\left[(e^{(k_{1}-k_{3}-k_{6})t}-e^{(k_{4}-k_{5}-k_{7})t})\left(k_{2}+2k_{3}\right)\right]}{\left(k_{1}-k_{3}-k_{4}+k_{5}-k_{6}+k_{7}\right)}\;\wedge$

 **A5**: $\forall t.\;D\left(t\right)=\frac{\left(2e^{-k_{8}t}\left(k_{2}+2k_{3}\right)k_{5}[\left(-1+e^{(k_{4}-k_{5}-k_{7}+k_{8})t}\right)k_{1}+k_{3}+k_{4}-k_{5}+k_{6}-k_{7}\right]}{\left(-k_{1}+k_{3}+k_{4}-k_{5}+k_{6}-k_{7}\right)\left(-k_{1}+k_{3}+k_{6}-k_{8}\right)\left(-k_{4}+k_{5}+k_{7}-k_{8}\right)}\;+$

   $\frac{\left(2e^{-k_{8}t}\left(k_{2}+2k_{3}\right)k_{5}[e^{(k_{1}-k_{3}-k_{6}+k_{8})t}\left(-k_{4}+k_{5}+k_{7}-k_{8}\right)+e^{(k_{4}-k_{5}-k_{7}+k_{8})t}\left(-k_{3}-k_{6}+k_{8}\right)\right]}{\left(-k_{1}+k_{3}+k_{4}-k_{5}+k_{6}-k_{7}\right)\left(-k_{1}+k_{3}+k_{6}-k_{8}\right)\left(-k_{4}+k_{5}+k_{7}-k_{8}\right)}\;\wedge$

 **A6:** real_derivative M(t) = k_6_ CSC(t) + k_7_ P(t) + k_8_ D(t)

 ⇒ entities_deriv_vec [CSC; P; D; M] t =

  transp (st_matrix (tumor_growth_model CSC P D M k_1_ k_2_ k_3_ k_4_ k_5_ k_6_ k_7_ k_8_ t))

     ∗ ∗ flux (tumor_growth_model CSC P D M k_1_ k_2_ k_3_ k_4_ k_5_ k_6_ k_7_ k_8_ t))

where the first two assumptions (A1-A2) ensure that the time evolution expressions of P and D do not contain any singularity (i.e., the value at the expression becomes undefined). The next three assumptions (A3-A5) provide the time evolution expressions for CSC, P and D, respectively. The last assumption (A6) is provided to discharge the subgoal characterizing the time-evolution of M (dead cells), which is of no interest and does not impact the overall analysis as confirmed by experimental evidences [20]. Finally, the conclusion of Theorem 2 is the equivalent reaction kinetic (ODE) model of the CSC based tumor growth model. To facilitate the verification process of the above theorem, we developed a simplifier, called KINETIC SIMP, which sufficiently reduces the manual reasoning interaction with the theorem prover. After the application of this simplifier, it only takes some arithmetic reasoning to conclude the proof of Theorem 2. More details about the verification process can be found on our project’s webpage [63].

The formal verification of the time-evolution of tumor cell types CSC, P and D in Theorem 2 can be easily used to formally derive the total population and volume of tumor cells. The derived time-evolution expression, verified in Theorem 2, can also be used to understand how the overall tumor growth model works. Moreover, potential drugs are usually designed using the variation of the kinetic rate constants, such as *k*_1_, *k*_2_⋯*k*_8_ in Theorem 2, to achieve the desired behavior of the overall tumor growth model and thus Theorem 2 can be utilized to study this behavior formally. On similar lines, the variation of these parameters is used to plan efficient therapeutic strategies for cancer patients and thus the formally verified result of Theorem 2 can aid in accurately performing this task.

#### Combined Zsyntax and Reaction kinetic based formal analysis of the tumor growth model

In this section, we consider another model for the growth of tumor cells and formally analyze it using both of our Zsyntax and Reaction kinetics formalizations, presented in the *Results* section of the paper.

**Pathway Leading to Death of CSC**

**Fig 5:**
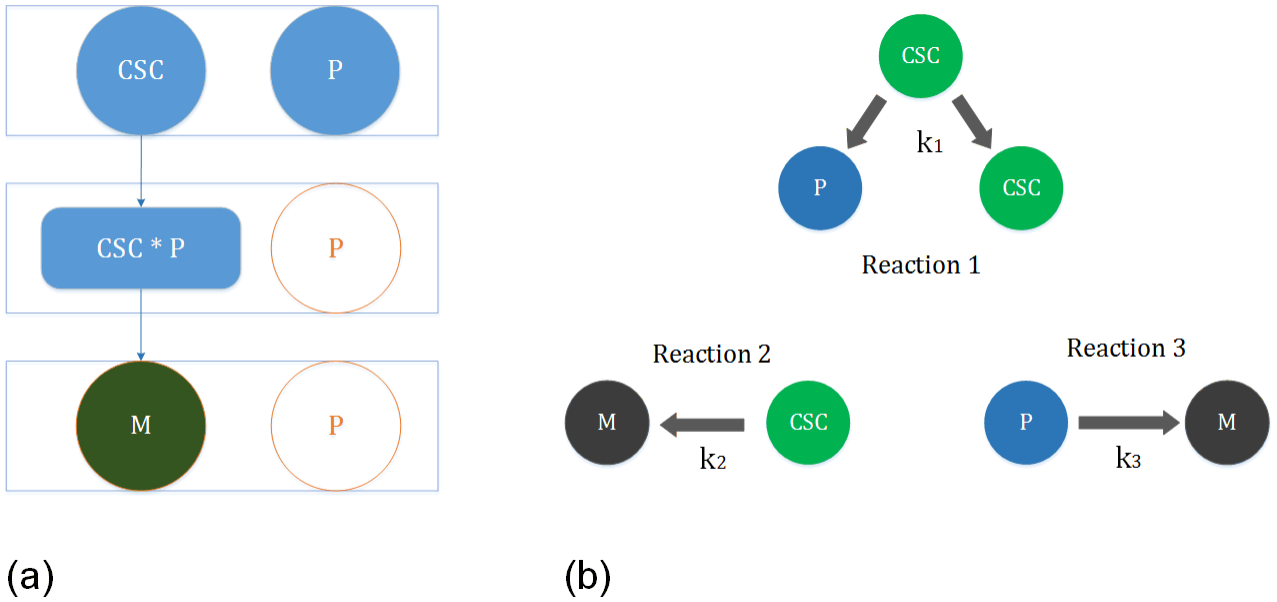
Case studies. (a) Reaction Representing the death of CSC (b) Another Model for the Growth of Tumor Cell.

The pathway leading to death of CSC is shown in Fig 5a. The green-colored circle represents the desired product, whereas, the blued-colored circles describe the chemical interactions in the pathway. We use our formalization of Zsyntax to deduce this pathway. In the classical Zsyntax format, the reaction of the pathway leading from CSC to its death can be represented by a theorem as CSC & P ⊢ M. Based on our formalization, it can be defined as follows:

**Theorem 3**. The Reaction of the Pathway Leading from CSC to its Death (M)

⊢ DISTINCT [CSC; P; M] ⇒

 zsyn_conjun_elimin (zsyn_deduct [[CSC];[P]]

  [([CSC],[[CSC;P]]);

   ([CSC;P],[[M]])]) [M] = [[M]]

In the above theorem, the first argument of the function zsyn_deduct represents the list of IA of molecules that are present at the start of the reaction, whereas the second argument is the list of valid EVFs for this reaction specified in the form of pairs and include the molecules (CSC, P, etc.). We use the HOL Light function DISTINCT to ensure that all molecule variables (from IA and EVFs) used in this theorem represent distinct molecules. Thus, the final list of molecules is deduced under these particular conditions using the function zsyn_deduct. Finally, if the molecule M is present in the post-reaction list of molecules, it will be obtained after the application of the function zsyn_conjun_elimin. We use the simplifier Z_SYNTAX_SIMP [63] to formally verify Theorem 3 automatically.

**Reaction Kinetic based Formal Analysis of a Tumor Growth based on CSC**

We perform the reaction kinetic based formal analysis of a tumor growth model, which is shown in Fig 5b. In this model, two types of events are considered: 1) maturation of CSCs into P cells; 2) death of all cell subtypes. All of these types of reactions are driven by different rate constants as shown in Fig 5b.

In the following, we provide the possible reactions in the considered tumor growth model:

1. A CSC can undergo asymmetric division (whereby one CSC gives rise to another CSC and a more differentiated P cell), i.e., $\text{CSC}\overset{{\text{k}}_{1}}{\to }\text{CSC}+\text{P}$.
2. All cellular subtypes can undergo cell death (M), i.e., $\text{CSC}\overset{{\text{k}}_{2}}{\to }\text{M}$, $\text{P}\overset{{\text{k}}_{3}}{\to }\text{M}$.

In order to reduce the complexity of the resulting model, only two subtypes of cells are considered: CSCs and transit amplifying progenitor cells (P) as shown in Fig 5b. Our main objective is to derive the mathematical expressions, which characterize the time evolution of CSC and P. Concretely, the values of these cells should satisfy the set of differential equations that arise in the kinetic model of the proposed tumor growth. Once the expressions of all cell types are known, the total number of tumor cells (*N*) in the human body can be computed by the formula *N*(*t*) = *CSC*(*t*) + *P*(*t*). We formalize the reaction kinetic based tumor growth model and verify the time evolution expressions for CSC and P that satisfy the general kinetic model. We formally represent this requirement in the following HOL Light theorem:

**Theorem 4.** Time Evolution Verification of a Tumor Growth Model

⊢ ∀ k_1_ k_2_ k_3_ CSC P M t.

 **A1:** (k_3_ −k_2_ ≠0) ∧

 **A2:** ∀t. CSC(t) = e^−k_2_ ∗ t^ ∧

 **A3**: $\forall t.\;P\left(t\right)=\frac{\left[\left(k_{3}-k_{2}-k_{1}\right)e^{-k_{3}t}+k_{1}e^{-k_{2}t}\right]}{\left(k_{3}-k_{2}\right)}\;\wedge$

 **A4:** real_derivative M(t) = k_2_ CSC(t) + k_3_ P(t)

 ⇒ entities_deriv_vec [CSC; P; M] t =

  transp (st_matrix (tumor_growth_rk_model CSC P M k_1_ k_2_ k_3_ t))

    ∗ ∗ flux (tumor_growth_rk_model CSC P M k_1_ k_2_ k_3_ t))

where the first assumption (A1) ensures that the time evolution expression of P does not contain any singularity. The next two assumptions (A2-A3) provide the time evolution expressions for CSC and P, respectively. The last assumption (A4) is provided to discharge the subgoal characterizing the time-evolution of M (dead cells), which is of no interest and does not impact the overall analysis as confirmed by experimental evidences [20]. Finally, the conclusion of Theorem 4 is the equivalent reaction kinetic (ODE) model of the CSC based tumor growth model. To facilitate the verification process of the above theorem, we use the KINETIC_SIMP simplifier, which sufficiently reduces the manual reasoning interaction with the theorem prover. After the application of this simplifier, it only takes some arithmetic reasoning to conclude the proof of Theorem 4. More details about the verification process can be found at [63].

## Discussion

Most of the existing research related to the formal analysis of the biological systems has been focussed on using model checking. However, this technique suffers from the inherent state-space explosion problem, which limits the scope of this success to systems where the biological entities can acquire only a small set of possible levels. Moreover, the underlying differential equations describing the reaction kinetics are solved using numerical approaches [69], which compromises the precision of the analysis. To the best of our knowledge, our work is the first one to leverage the distinguishing features of interactive theorem proving to reason about the solutions to system biology problems. We consider the concentration of the species of the biological systems in reaction kinetic based formal analysis as a continuous variable. Besides formalizing Zsyntax and the reaction kinetics of commonly used biological pathways, we also formally verified their classical properties. This verification guarantees the soundness and the correctness of our formal definitions. It also enables us to conduct formal analysis of real-world case studies. In order to illustrate the practical effectiveness of our formalization, we presented the automatic Zsyntax based formal analysis of pathway leading to TP53 Phosphorylation and a pathway leading to the death of CSCs in the tumor growth model, and reaction kinetics based analysis of the tumor growth model. Our source code is available online [63] and can be used by other biologists and computer scientists for further applications and experimentation.

The distinguishing feature of our framework is the ability to deductively reason about biological systems using both Zsyntax and reaction kinetics. The soundness of interactive theorem proving ensures the correct application of EVFs or the simplification process as there is no risk of human error. The involvement of computers in the formal reasoning process of the proposed approach makes it more scalable than the analysis presented in *Boniolo et al.*’s and *Molina-Pena et al.*’s paper [13, 20], which is based on traditional paper-and-pencil based analysis technique. Another key benefit of the reported work is the fact that the assumptions of these formally verified theorems are guaranteed to be complete, due to the soundness of the underlying analysis methods, and thus enables us to get a deep understanding about the conditions and constraints under which a Zsyntax and reaction kinetics based analysis is performed. Also, we have verified generic theorems with universally quantified variables and thus the analysis covers all possibilities. Similarly, in the case of reaction kinetics based analysis, the theorems have been verified for arbitrary values of parameters, such as *k*_1_ and *k*_2_, which is not possible in the case of simulation where these expressions are tested for few samples of such parameters. A major limitation of higher-order logic theorem proving is the manual guidance required in the formal reasoning process. But we have tried to facilitate this process by formally verifying frequently used results, such as, simplification of vector summation manipulation and verification of flux vectors and stoichiometric matrices for each of the reaction schemes, and providing automation where possible. For example, we have developed two simplifiers, namely Z_SYNTAX_SIMP and KINETIC_SIMP, that have been found to be very efficient in automatically simplifying most of the Zsyntax or reaction kinetic related proof goals, respectively. In the first case study, the simplifier Z_SYNTAX_SIMP allowed us to automatically verify the theorem representing the reaction of the pathway leading to TP53 Phosphorylation. Similarly, in the second case study, i.e., time evolution verification of the tumor growth model, the simplifier KINETIC_SIMP significantly reduced the manual interaction and the proof concluded using this simplifier and some straightforward arithmetic reasoning. These simplifiers are also used to automate the verification process of the third case study, i.e., the automatic verification of the theorem representing the reaction of the pathway leading to the death of CSC and a significant simplification of the verification of the theorem representing the time evolution for the growth of the tumor cell.

In future, we plan to conduct the sensitivity and steady state analysis [14] of biological networks that is mainly based on reaction kinetics. We also plan to integrate Laplace [70] and Fourier [71] transforms formalization in our framework that can assist in finding analytical solutions of the complicated ODEs.

## Supporting information

S1 Report Technical report. (PDF)

S1 Formalization Formalization of Zsyntax. (ML)

S2 Formalization Formalization of reaction kinetics. (ML)
