# Supplementary material for: Formal reasoning about systems biology using theorem proving
Source: PLoS One. 2017 Jul 3;12(7):e0180179. doi: 10.1371/journal.pone.0180179 (PMC5495343; doi:10.1371/journal.pone.0180179)
Supplement: S1 Report — (PDF) [file pone.0180179.s001.pdf]

Formal Reasoning about Systems Biology using Theorem Proving

Adnan Rashid*, Osman Hasan*, Umair Siddique** and Sofiène Tahar**
*School of Electrical Engineering and Computer Science (SEECS),
National University of Sciences and Technology (NUST), Islamabad, Pakistan
{adnan.rashid,osman.hasan}@seecs.nust.edu.pk
**Department of Electrical and Computer Engineering,
Concordia University, Montreal, Canada
{muh_sidd,tahar}@ece.concordia.ca

Abstract

System biology provides the basis to understand the behavioral properties of complex biological organisms at different levels of abstraction. Traditionally, the analysis of systems biology based models of various diseases has been carried out by paper-and-pencil based proofs and simulations. However, these methods cannot provide accurate analysis, which is a serious drawback for the safety-critical domain of human medicine. In order to overcome these limitations, we propose a framework to formally analyze biological networks and pathways. In particular, we formalize the notion of reaction kinetics in higher-order logic and formally verify some of the commonly used reaction based models of biological networks using the HOL Light theorem prover. Furthermore, we have ported our earlier formalization of Zsyntax, i.e., a deductive language for reasoning about biological networks and pathways, from HOL4 to the HOL Light theorem prover to make it compatible with the above-mentioned formalization of reaction kinetics. To illustrate the usefulness of the proposed framework, we present the formal analysis of three case studies, i.e., the pathway leading to TP53 Phosphorylation, the pathway leading to the death of cancer stem cells and the tumor growth based on cancer stem cells, which is used for the prognosis and future drug designs to treat cancer patients.

Keywords: Systems Biology, Molecular Pathways, Reaction Kinetics, Automated Reasoning, Theorem Proving

1 Introduction

The discovery and designing of effective drugs for infectious and chronic biological diseases, like cancer and cerebral malaria, require a deep understanding of behaviorial and structural characteristics of involved biological entities (e.g., cells, molecules and enzymes). Traditional approaches, which rely on verbal and personal intuitions without concrete logical explanations of biological phenomena, often fail to provide a complete understanding of the behavior of such diseases, mainly due to the underlying complex interactions of molecules connected through a chain of reactions. *Systems biology* [1] overcomes these limitations by integrating mathematical modeling and high-speed computing machines in the understanding of biological processes and thus provides the ability to predict the effect of potential drugs for the treatment of chronic diseases. System biology is extensively used to construct models of biological processes in the form of networks or pathways, such as protein-protein interaction networks and signaling pathways [2]. The analysis of these biological networks, usually referred to as biological regulatory networks (BRNs) or gene regulatory networks (GRNs) [3], is primarily based on the principles of molecular biology and plays a vital role in investigating the treatment of various human infectious diseases and future drug design targets. For example, the analysis of BRNs has been recently used to predict treatment decisions for sepsis patients [4].

Traditionally, biologists analyze biological organisms (or different diseases) using wet-lab experiments [5, 6]. These experiments, despite being very slow and expensive, do not ensure accurate results due to the inability to accurately characterize the complex biological processes in an experimental setting. One of the alternatives for deducing molecular reactions include paper-and-pencil proof methods (e.g. using Boolean modeling [7] or kinetic logic [8]). But the manual proofs in paper-and-pencil proof methods, become quite tedious for large systems, where the calculation of unknown parameters takes several hundred proof steps, and are thus prone to human errors. Other alternatives to the above-mentioned methods are computer-based techniques (e.g. Petri nets [9] and model checking [10]) for analyzing molecular biology problems. Petri nets is a graph based technique [11] for analyzing system properties. In model checking, a system is modeled in the form of state space or automata and the intended properties of the system are verified in a model checker by a rigorous state exploration of the

system model. The computer-based methods have shown very promising results in many applications of molecular biology [12, 13]. However, the graph or state based nature of the models in these methods allow us to describe some specific areas of molecular biology only [14]. Moreover, the inherent state-space explosion problem of model checking [15] limits the scope of its success only to systems where the biological entities can acquire a small set of possible levels.

Theorem proving [16] is a widely used formal methods technique that allows to construct a computer-based mathematical model of the given system and then verify its properties using deductive reasoning. The foremost requirement for conducting the theorem proving based analysis of any system is to formalize the mathematical or logical foundations required to model and analyze that system in an appropriate logic. There have been several attempts to formalize the foundations of molecular biology. For example, the earliest axiomatization even dates back to 1937 [17] and other efforts related to the formalization of biology are presented by *Zanardo et al.*[18] and *Rizzotti et al.* [19]. But these formalizations are paper-and-pencil based and thus cannot be utilized to formally reason about molecular biology problems within a theorem prover integration. Recent formalizations of molecular biology, based on K-Calculus [20] and π-Calculus [21], also include some formal reasoning support for biological systems. Another interesting approach is to model signal transduction pathways using the pathway logic [22], which is based on rewriting logic. But, the understanding and utilization of these techniques is generally very cumbersome for a working biologist [23].

Zsyntax [14] is another recently proposed formal language that supports modeling and logical deductions about any biological process. The main strength of Zsyntax is its biologist-centered nature as its operators and inference rules have been designed in such a way that they are easily understandable by the biologists. However, Zsyntax does not support specifying the temporal information associated with biological processes. *Reaction kinetics* [24] caters for this limitation by providing the basis to understand the time evolution of molecular populations involved in a biological network. This approach is based on the set of first-order ordinary differential equations (ODEs) also called *reaction rate equations* (RREs). Most of these equations are non-linear in nature and difficult to analyze but provide very useful insights which can be used for prognosis and drug predictions. Traditionally, logical deductions about biological processes, expressed in Zsyntax, are done manually based on the paper-and-pencil based approach. Similarly, the analysis of RREs is performed by either paper-and-pencil based proofs or numerical simulation. However, both methods suffer from the inherent incompleteness of numerical methods and error-proneness of manual proofs. We believe that these issues cannot be ignored considering the critical nature of this analysis due to the involvement of human lives. Moreover, biological experiments based on erroneous parameters derived by above-mentioned approaches can result in the loss of time and money due to the slow nature of wet-lab experiments and the cost associated with the chemicals and measurement equipments.

In this report, we propose to develop a formal reasoning support for system biology to analyze complex biological systems within the sound core of a theorem prover and thus provide accurate analysis results in this safety-critical domain. In our recent work [25], we developed a formal deduction framework for reasoning about molecular reactions by formalizing the Zsyntax language in the HOL4 theorem prover [26] . In particular, we formalized the logical operators and inference rules of Zsyntax in higher-order logic. We then built upon these formal definitions to verify two key behavioral properties of Zsyntax based molecular pathways [27, 28]. However, it was not possible to reason about biological models based on reaction kinetics due to the unavailability of the formal notions of reaction rate equations (a set of coupled differential equations) in higher-order logic. In order to broader the horizons of formal reasoning about system biology, this report presents a formalization of reaction kinetics along with the development of formal models of generic biological pathways without the restriction on the number of molecules and corresponding interconnections. Furthermore, we formalize the transformation, which is used to convert biological reactions into a set of coupled differential equations. This step requires multivariate calculus (e.g., vector derivative, matrices, etc.) formalization in higher-order logic, which is not available in HOL4 and therefore we have chosen to leverage upon the rich multivariable libraries of the HOL Light theorem prover [29] to formalize the above mentioned notions and verify the reactions kinetics of some generic molecular reactions. To make the formalization of Zsyntax[25] consistent with the formalization of reaction kinetics in HOL Light, we ported all of the HOL4 formalization of Zsyntax to HOL Light as part of our current work.

The rest of the report is organized as follows: Section 2 presents the theorem proving based proposed framework to formally reason about the system biology. Section 3 provides an introduction to the preliminaries of Zsyntax, reaction kinetics and higher-order-logic theorem proving. We present the formalization of Zsyntax along with the verification of important properties in Section 4. This is followed by the formalization of reaction kinetics and the verification of some general reaction rate equations based models in Section 5. The illustration of our proposed framework is presented in Section 6, where we describe the formal analysis of a molecular reaction representing the TP53 Phosphorylation, a molecular reaction representing the pathway leading to the death of cancer stem cells and the verification of a tumor growth model based on cancer stem cells. Finally, we conclude our work and highlight some future directions in Section 7.

2 Proposed Framework

The proposed theorem proving based formal reasoning framework for system biology, depicted in Figure 1, allows to formally deduce the complete pathway from any given time instance and model and analyze the ordinary differential equations (ODEs) corresponding to a kinetic model for any molecular reaction. For this purpose, the framework builds upon the existing higher-order-logic formalizations of Lists, Pairs, Vectors, and Calculus.

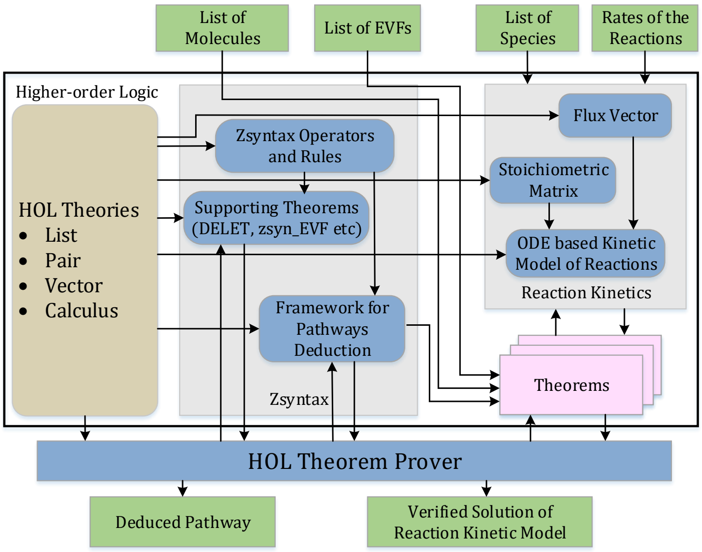

Figure 1: Proposed Framework

The two main rectangles in the higher-order logic block present the foundational formalizations developed to facilitate the formal reasoning about the Zsyntax based pathway deduction and the reaction kinetics. In order to perform the Zsyntax based molecular pathway deduction, we first formalize the functions representing the logical operators and inference rules of Zsyntax in higher-order logic and verify some supporting theorems from this formalization. This formalization can then be used along with a list of molecules and a list of *Empirically Valid Formulae* (EVFs) to formally deduce the pathway for the given list of molecules and provide the result as a formally verified theorem using HOL Light. Similarly, we have formalized the flux vectors and stoichiometric matrices in higher-order-logic. These foundations can be used along with a given list of species and the rate of the reactions to develop a corresponding ODEs based kinetic reactions model. The solution to this ODE can then be formally verified as a theorem by building upon the existing formalization of Calculus theories.

The distinguishing characteristics of the proposed framework include the usage of deductive reasoning to derive the deduced pathways and solutions of the reaction kinetic models. Thus, all theorems are guaranteed to be correct and explicitly contain all the required assumptions.

3 Preliminaries

In this section, we provide a brief introduction to Zsyntax, reaction kinetics and higher-order-logic theorem proving. The main intent is to review the main concepts and notations used in this report.

3.1 Zsyntax

Zsyntax [14] exploits the analogy between biological processes and logical deduction. Some of the key features of Zsyntax are: 1) the ability to express molecular reactions in a mathematical way; 2) heuristic nature, i.e., if the conclusion of a reaction is known, then one can deduce the missing data from the initialization data; 3) computer implementable semantics. Zsyntax consists of the following three operators:

Z-Interaction: The interaction of two molecules is expressed by the Z-Interaction ($*$) operator. In biological reactions, Z-interaction is not associative.

Z-Conjunction: The aggregate of same or different molecules (not necessarily interacting with each other) is formed using the Z-Conjunction (&) operator. Z-Conjunction is fully associative.

Z-Conditional: A path from A to B under the condition C is expressed using the Z-Conditional ($\rightarrow$) operator as: $A \rightarrow B$ if there is a C that allows it.

Zsyntax supports four inference rules, given in Table 1, that play a vital role in deducing the outcomes of biological reactions:

Table 1: Zsyntax Inference Rules

**Inference Rules**	**Definition**
Elimination of Z-conditional($\rightarrow$E)	`if` $\mathtt{C} \vdash (\mathtt{A} \rightarrow \mathtt{B})$ `and` $(\mathtt{D} \vdash \mathtt{A})$ `then` $(\mathtt{C\&D} \vdash \mathtt{B})$
Introduction of Z-conditional($\rightarrow$I)	$\mathtt{C\&A} \vdash \mathtt{B}$ `then` $\mathtt{C} \vdash (\mathtt{A} \rightarrow \mathtt{B})$
Elimination of Z-conjunction(&E)	$\mathtt{C} \vdash (\mathtt{A\&B})$ `then` $(\mathtt{C} \vdash \mathtt{A})$ `and` $(\mathtt{C} \vdash \mathtt{B})$
Introduction of Z-conjunction(&I)	$(\mathtt{C} \vdash \mathtt{A})$ `and` $(\mathtt{D} \vdash \mathtt{B})$ `then` $(\mathtt{C\&D}) \vdash (\mathtt{A\&B})$

Besides the regular formulas that can be derived based on the above-mentioned operators and inference rules, Zsyntax also makes use of *Empirically Valid Formulae* (EVF). These EVFs basically represent the non-logical axioms of molecular biology and are assumed to be validated empirically in the lab.

It has been shown that any biological reaction can be mapped and their final outcomes can be derived using the above-mentioned three operators and four inference rules [14]. For example, consider a scenario in which three molecules A, B and C react with each other to yield another molecule Z. This can be represented as a Zsyntax theorem as follows:

$$\mathtt{A \ \& \ B \ \& \ C} \vdash \mathtt{Z}$$

The Z-Conjunction operator & is used to represent the given aggregate of molecules and then the inference rules from Table 1 are applied on these molecules along with some EVFs (chemical reactions verified in laboratories) to obtain the final product Z. For the above example, these EVFs could be:

$$\mathtt{A} * \mathtt{B} \rightarrow \mathtt{X} \text{ and } \mathtt{X} * \mathtt{C} \rightarrow \mathtt{Z}$$

which means that A will react with B to yield X and X in return will react with C to yield the final product Z.

3.2 Reaction Kinetics

Reaction kinetics [30] is the study of rates at which biological processes interact with each other and how the corresponding processes are affected by these reactions. The rate of a reaction provides the information about the evolution of the concentration of the species (e.g., molecules) over time. A process is basically a chain of reactions, called pathway, and the investigation about the rate of a process implies the rate of these pathways. Generally, biological reactions can be either irreversible (unidirectional) or reversible (bidirectional).

In order to analyze a biological process, we need to know its kinetic reaction based model, which comprises a set of m species, $X = \{X_1, X_2, X_3, \ldots, X_m\}$ and a set of n reactions, $R = \{R_1, R_2, R_3, \ldots, R_n\}$.

An irreversible reaction R_j, $\{1 \leq j \leq n\}$ can generally be written as follows:

$$R_j : s_{1,j}X_1 + s_{2,j}X_2 + \ldots + s_{m,j}X_m \xrightarrow{\mathrm{k_j}} \acute{s}_{1,j}X_1 + \acute{s}_{2,j}X_2 + \ldots + \acute{s}_{m,j}X_m \tag{1}$$

Similarly, a reversible reaction R_j, $\{1 \leq j \leq n\}$ can be described as follows:

$$R_j : s_{1,j}X_1 + s_{2,j}X_2 + \ldots + s_{m,j}X_m \underset{k_j{}^r}{\overset{k_j{}^f}{\rightleftarrows}} \acute{s}_{1,j}X_1 + \acute{s}_{2,j}X_2 + \ldots + \acute{s}_{m,j}X_m \tag{2}$$

The coefficients $s_{1,j}, s_{2,j}, \ldots, s_{m,j}, \acute{s}_{1,j}, \acute{s}_{2,j}, \ldots, \acute{s}_{m,j}$ are the non-negative integers and represent the stoichiometries of the species taking part in the reaction. The non-negative integer k_j is the kinetic rate constant of the irreversible reaction. The non-negative integers $k_j{}^f$ and $k_j{}^r$ are the forward and reverse kinetic rate constants of the reversible reaction [31].

A reversible reaction can be divided into two irreversible reactions with the forward kinetic rate constant as the kinetic rate constant of the first reaction and the reverse kinetic rate constant as the kinetic rate constant of the second reaction as given below:

$$R_j{}^{(1)} : s_{1,j}X_1 + s_{2,j}X_2 + \ldots + s_{m,j}X_m \xrightarrow{k_j{}^{\mathrm{f}}} \acute{s}_{1,j}X_1 + \acute{s}_{2,j}X_2 + \ldots + \acute{s}_{m,j}X_m$$

$$R_j{}^{(2)} : \acute{s}_{1,j}X_1 + \acute{s}_{2,j}X_2 + \ldots + \acute{s}_{m,j}X_m \xrightarrow{k_j{}^{\mathrm{r}}} s_{1,j}X_1 + s_{2,j}X_2 + \ldots + s_{m,j}X_m$$

The dynamic behaviour of the biochemical systems is described by the set of ordinary differential equations (ODEs) as a counterpart to the above mentioned reaction based models. The evolution of the system is captured by analyzing the change in the concentration of the species (i.e., time derivatives). Mathematically, this can be described as follows:

$$\frac{d[X_i]}{dt} = \sum_{j=1}^{n} n_{i,j} v_j \tag{3}$$

where $n_{i,j}$ is the stoichiometric coefficient of the molecular species X_i in reaction R_j (i.e., $n_{i,j} = \acute{s}_{i,j} - s_{i,j}$). The parameter v_j represents the flux of the reaction R_j.

For all the reactions from 1 to n, it becomes a flux vector as $v = (v_1, v_2, \ldots, v_n)^T$ and the system of ODEs can be written in the vectorial form as follows:

$$\frac{d[\mathbf{X}]}{dt} = N\mathbf{v} \tag{4}$$

where $[\mathbf{X}] = (X_1 X_2, \ldots, X_n)^T$ is vector of the concentration of all of the species participating in the reaction and N is the stoichiometric matrix of order $m \times n$.

For example, consider the following simple example with two reactions:

$$A \xrightarrow{\mathrm{k}} B \qquad\qquad A + 2B \underset{k^r}{\overset{k^f}{\rightleftarrows}} C$$

The first reaction is irreversible with reaction kinetic rate constant k and the second is a reversible reaction with k^f and k^r as the forward and reverse reaction kinetic rate constants, respectively. The vector for the concentration of species is $([A], [B], [C])^T$ and the flux vector is $v = (v_1, v_2)^T$, where v_1 is the flux of first reaction and v_2 is the flux of second reaction. The above reaction model can be written as follows:

$$A + 0B + 0C \xrightarrow{\mathrm{k}} 0A + B + 0C \qquad\qquad A + 2B + 0C \underset{k^r}{\overset{k^f}{\rightleftarrows}} 0A + 0B + C$$

Now we can derive the stoichiometric matrix for above reaction as follows:

$$N = \begin{bmatrix} 0-1 & 0-1 \\ 1-0 & 0-2 \\ 0-0 & 1-0 \end{bmatrix} = \begin{bmatrix} -1 & -1 \\ 1 & -2 \\ 0 & 1 \end{bmatrix}$$

The ODE model for the above reaction model is given as follows:

$$\begin{bmatrix} \frac{d[A]}{dt} \\ \frac{d[B]}{dt} \\ \frac{d[C]}{dt} \end{bmatrix} = \begin{bmatrix} -1 & -1 \\ 1 & -2 \\ 0 & 1 \end{bmatrix} \cdot \begin{bmatrix} v_1 \\ v_2 \end{bmatrix} \tag{5}$$

The flux of a reaction can be computed by the law of mass action [24], which states that the rate (also called flux) of a reaction is proportional to the concentration of the reactant raised its stoichiometry. The stoichiometry of A is 1 in the first reaction and thus the rate for the irreversible reaction is $v_1 = k[A]$

The reversible reaction can be written as two irreversible reactions, so the rate/flux of the above reversible reaction is obtained by taking the differences of the fluxes of the two irreversible reactions as follows:

$$v = v_f - v_r = k^f[A][B]^2 - k^r C \tag{6}$$

where v_f and v_r represent the forward reaction rate and the reverse reaction rate, respectively, of the given reversible reaction. From the above description, it is clear that an ODE model of the biochemical system explains the concentration of the species which varies over time as a function of reaction flux which further depends on the kinetics of the reaction.

3.3 Higher-order-Logic Theorem Proving and HOL Light Theorem Prover

Theorem proving is concerned with the construction of mathematical proofs by a computer program using axioms and hypothesis. Theorem proving systems are widely used in software and hardware verification [32, 33, 34]. For example, hardware designer can prove different properties of a digital circuit by using some predicates to model the circuits model. Similarly, a mathematician can prove the transitivity property for real numbers using the axioms of real number theory. The language of these mathematical theorems or conjectures is logic, which can be propositional logic, first-order logic or higher-order logic depending upon the expressibility requirement. For example, the use of higher-order logic is advantageous over first-order logic in terms of the availability of additional quantifiers and its high expressiveness.

A theorem prover is a software in which formal reasoning about the correctness of mathematical theorems can be conducted with as much accuracy as pencil-and-paper, but with the precise control of the computer which ensures that no mathematical mistake is involved. For example, a theorem prover does not allow us to conclude that "$\frac{x}{x} = 1$" unless it is first proved or assumed that $x \neq 0$. This is achieved by defining a precise syntax of the mathematical sentences that can be input in the software. The designer of the theorem prover also provides some axioms and inference rules which are the only ways to prove a sentence correct. This purely deductive aspect provides the guarantee that every sentence proved in the system is true (in particular, there is no approximation like in computer algebra systems). When a mathematical or physical theory is expressed inside a theorem prover, we say that it is formalized.

Based on the decidability or undecidability of the underlying logic, theorem proving can be done automatically or interactively. Propositional logic is decidable and thus the sentences expressed in this logic can be automatically verified whereas higher-order logic is undecidable and thus the theorem proving about sentences, expressed in higher-order logic, have to be verified by providing guidance from its user in an interactive way.

HOL Light [29] is an interactive theorem proving environment for the construction of mathematical proofs in higher-order logic. A theorem is a formalized statement that may be an axiom or could be deduced from already verified theorems by an inference rule. A theorem consists of a finite set Ω of Boolean terms called the assumptions and a Boolean term S called the conclusion. For example, "$\forall x. x \neq 0 \Rightarrow \frac{x}{x} = 1$" represents a theorem in HOL Light. A HOL Light theory consists of a set of types, constants, definitions, axioms and theorems. HOL Light theories are organized in a hierarchical fashion and theories can inherit the types, constants, definitions and theorems of other theories as their parents. In this report, we make use of the HOL Light theories of Boolean variables, real numbers, transcendental functions, lists and multivariate analysis. In fact, one of the primary motivations of selecting the HOL Light theorem prover for our work was to benefit from these built-in mathematical theories. The proofs in HOL Light are based on the concept of a tactic that breaks goals into simple subgoals. There are many automatic proof procedures and proof assistants [35] available in HOL Light which help the user in directing the proof to the end.

Table 2 provides the mathematical interpretations of some frequently used HOL Light symbols and functions used in this report.

4 Formalization of Zsyntax

We model the molecules as variables of arbitrary data types (α) in our formalization of Zsyntax [28]. A list of molecules (α *list*) represents the Z-Interaction or a molecular reaction among the elements of the list. The Z-Conjunction operator forms a collection of non-reacting molecules and can now be formalized as a list of list of molecules (α *list list*). This data type allows us to apply the Z-Conjunction operator between individual molecules (a list with a single element) or multiple interacting molecules (a list with multiple elements). The Z-Conditional operator is used to update the status of molecules, i.e., generate a new set of molecules based on the available EVFs. Each EVF is modeled in our formalization as a pair (α *list* # α *list list*) where the first element is a list of molecules (α *list*) indicating the reacting molecules and the second element is a list of list of molecules (α *list list*) indicating the resulting set of molecules after the reaction between the molecules of the first element of the pair has taken place. A collection of EVFs is represented as a list of EVFs ((α *list* # α *list list*)*list*) in our formalization.

The elimination of Z-Conditional rule is the same as the elimination of implication rule (Modus Ponens) in propositional logic and thus it can be directly handled by the theorem prover simplification and rewriting rules. Similarly, the introduction of Z-Conditional rule can also be inferred from the rules of propositional logic and can be handled by the theorem prover without the introduction of a new inference rule. The elimination of the Z-Conjunction rule allows us to infer the presence of a single molecule from an aggregate of inferred molecules. This rule is usually applied at the end of the reaction to check if the desired molecule has been obtained. Based on our data types, described above, this rule can be formalized by a function that returns a particular molecule

Table 2: HOL Light Symbols and Functions

**HOL Symbol**	**Standard Symbol**	**Meaning**
/\	and	Logical *and*
\/	or	Logical *or*
~	not	Logical *negation*
`T`	true	Logical true value
`F`	false	Logical false value
==>	$\longrightarrow$	Implication
<=>	=	Equality in Boolean domain
!x.t	$\forall x.t$	for all $x : t$
λx.t	$\lambda x.t$	Function that maps x to $t(x)$
`HD L`	*head*	Head element of list L
`TL L`	*tail*	Tail of list L
`EL n L`	*element*	n^{th} element of list L
`MEM a L`	*member*	True if a is a member of list L
`LENGTH L`	*length*	Length of list L
`CONS`	*CONS*	Adds a new element to the top of the list
`APPEND`	*Append*	Merges two lists
`FST`	fst (a, b) = a	First component of a pair
`SND`	snd (a, b) = b	Second component of a pair
`num`	$\{0, 1, 2, \ldots\}$	Positive Integers data type
`real`	All Real numbers	Real data type
`suc n`	$(n + 1)$	Successor of natural number
`exp x`	e^x	Exponential function (real-valued)
`sqrt x`	$\sqrt{x}$	Square root function
`abs x`	$\|x\|$	Absolute function
&a	$\mathbb{N} \to \mathbb{R}$	Typecasting from Integers to Reals
`A**B`	$[A][B]$	Matrix-Matrix or Matrix-Vector multiplication

from a list of molecules:

Definition 4.1. Elimination of Z-Conjunction Rule
$\vdash$ ∀ `l x. zsyn_conjun_elimin l x =` **if** `MEM x l` **then** `[x] else l`

The function `zsyn_conjun_elimin` has the data type (α *list* $\to \alpha \to \alpha$ *list*). The above function returns the given element as a single element in a list if it is a member of the given list. Otherwise, it returns the argument list as it is.

The introduction of Z-Conjunction rule along with Z-Interaction allows us to perform a reaction between any of the available molecules during the experiment. Based on our data types, this rule is equivalent to the append operation of lists.

Definition 4.2. Introduction of Z-Conjunction and Z-Interaction
$\vdash$ ∀ `l x y. zsyn_conjun_intro l x y = CONS (FLAT [EL x l; EL y l]) l`

The above definition has the data type (α *list list* $\to$ *num* $\to$ *num* $\to$ α *list list*). The functions `FLAT` and `EL` are used to flatten a list of lists to a single list and return a particular element of a list, respectively. Thus, the function `zsyn_conjun_intro` takes a list `l` and appends the list of two of its elements `x` and `y` on its head.

Based on the laws of stoichiometry [14], the reacting molecules using the Z-Conjunction operator have to be deleted from the aggregate of molecules. The following function represents this behavior in our formalization:

Definition 4.3. Reactants Deletion

```
⊢ ∀ l x y.  zsyn_delet l x y = if x > y
                then delet (delet l x) y else delet (delet l y) x
```

Here the function `delet l x` deletes the element at index `x` of the list `l` and returns the updated list as follows:

Definition 4.4. Element Deletion

```
⊢ ∀ l.  delet l 0 = TL l ∧ ∀ l y.  delet l (y + 1) = CONS (HD l) (delet (TL l) y)
```

Thus, the function `zsyn_delet l x y` deletes the xth and y^{th} elements of the given list `l`. We delete the higher indexed element before the lower one in order to make sure that the first element deletion does not effect the index of the second element that is required to be deleted. The above data types and definitions can be used to formalize any molecular pathway (which is expressible using Zsyntax) and reason about its correctness within the sound core of the higher-order logic theorem prover.

Our main objective is to develop a framework that accepts a list of initial molecules and possible EVFs and allows the user to formally deduce the final outcomes of the corresponding biological experiment. In this regard, we first develop a function that compares a particular combination of molecules with all the EVFs and upon finding a match introduces the newly formed molecule in the initial list and deletes the consumed instances.

Definition 4.5. EVF Matching

```
⊢ ∀ l e x y.
zsyn_EVF l e 0 x y =
    if FST (EL 0 e) = HD l
      then (T,zsyn_delet (APPEND (TL l) (SND (EL 0 e))) x y
      else (F,TL l) ∧
  ∀ l e p x y.
zsyn_EVF l e (p + 1) x y =
    if FST (EL (p + 1) e) = HD l
      then (T,zsyn_delet (APPEND (TL l) (SND (EL (p + 1) e))) x y
      else zsyn_EVF l e p x y
```

The data type of the function `zsyn_EVF` is: (α *list list* $\rightarrow$ (α *list* # α *list list*) *list* $\rightarrow$ *num* $\rightarrow$ *num* $\rightarrow$ *num* $\rightarrow$ *bool* # α *list list*). The function `LENGTH` returns the length of a list. The function `zsyn_EVF` takes a list of molecules `l` and recursively checks its head, or the top most element, against all elements of the EVF list `e`. If there is no match, then the function returns a pair with its first element being false (`F`), indicating that no match occurred, and the second element equals the tail of the input list `l`. Otherwise, if a match is found then the function removes the head of initial list `l` with the second element of the EVF pair is added to the end of this modified list and deletes the matched elements from the initial list as these elements have already been consumed. This modified list is then returned along with a true (`T`) value, which acts as a flag to indicate an element replacement.

Next, in order to deduce the final outcome of the experiment, we have to call the function `zsyn_EVF` recursively by placing all the possible combinations of the given molecules at the head of list `l` one by one. This can be done as follows:

Definition 4.6. Recursive Function to model the argument `y` in Definition 4.5

```
⊢ ∀ l e x.  zsyn_recurs1 l e x 0 =
   zsyn_EVF (zsyn_conjun_intro l x 0) e (LENGTH e - 1) x 0 ∧
  ∀ l e x y.  zsyn_recurs1 l e x (y + 1) =
    if FST (zsyn_EVF (zsyn_conjun_intro l x (y + 1)) e (LENGTH e - 1) x (y + 1)) ⇔ T
      then zsyn_EVF (zsyn_conjun_intro l x (y + 1)) e (LENGTH e - 1) x (y + 1)
      else zsyn_recurs1 l e x y
```

Definition 4.7. Recursive Function to model the argument x in Definition 4.5

```
⊢ ∀ l e y.  zsyn_recurs2 l e 0 y =
    if FST (zsyn_recurs1 l e 0 y) ⇔ T
      then (T,SND (zsyn_recurs1 l e 0 y))
      else (F,SND (zsyn_recurs1 l e 0 y)) ∧
  ∀ l e x y.  zsyn_recurs2 l e (x + 1) y =
    if FST (zsyn_recurs1 l e (x + 1) y) ⇔ T
      then (T,SND (zsyn_recurs1 l e (x + 1) y))
      else zsyn_recurs2 l e x (LENGTH l - 1)
```

Both the above functions have the same data type, i.e, (α *list list* $\rightarrow$ (α *list* $\#\alpha$ *list list*) *list* $\rightarrow$ *num* $\rightarrow$ *num* $\rightarrow$ *num* $\rightarrow$ *bool* $\#$ α *list list*). The function zsyn_recurs1 calls the function zsyn_EVF after appending the combination of molecules indexed by variables x and y using the introduction of Z-Conjunction rule. Thus, the new combination is always placed on the top of the list, which is passed as the variable l to the function zsyn_EVF. Moreover, we provide the length of the EVF list (LENGTH e - 1) as the variable p of the function zsyn_EVF so that the new combination is compared to all the entries of the EVF list. It is also important to note that the function zsyn_recurs1 terminates as soon as a match in the EVF list is found. This function also returns a flag indicating the status of this match as the first element of its output pair. The second function zsyn_recurs2 checks this flag and if it is found to be true (meaning that a match in the EVF list is found) then it terminates by returning the output list of the function zsyn_recurs1. Otherwise, it continues with all the remaining values of the variable x recursively. In the first case, i.e., when a match is found, the above two functions have to be called all over again with the new list. These iterations will continue until there is no match found in the execution of functions zsyn_recurs1 and zsyn_recurs2. This overall behaviour can be modelled by the following recursive function:

Definition 4.8. Final Recursion Function for Zsyntax

```
⊢ ∀ l e x y.  zsyn_deduct_recurs l e x y 0 = (T,l) ∧
  ∀ l e x y q.  zsyn_deduct_recurs l e x y (q + 1) =
    if FST (zsyn_recurs2 l e x y) ⇔ T
      then zsyn_deduct_recurs (SND (zsyn_recurs2 l e x y)) e
          (LENGTH (SND (zsyn_recurs2 l e x y)) - 1) (LENGTH (SND (zsyn_recurs2 l e x y)) - 1) q
      else (T,SND (zsyn_recurs2 l e (LENGTH l - 1) (LENGTH l - 1)))
```

The function zsyn_deduct_recurs also has the same data type as the above mentioned recursion functions. The variable of recursion in this function should be assigned a value that is greater than the total number of EVFs so that the application of none of the EVF is missed to guarantee correct operation. Similarly, the variables x and y above should be assigned the values of (LENGTH l - 1) to ensure that all the combinations of the list l are checked against the elements of the list of EVFs. Thus, the final deduction function for Zsyntax can be expressed in HOL Light as follows:

Definition 4.9. Final Deduction Function for Zsyntax

```
⊢ ∀ l e.  zsyn_deduct l e = SND (zsyn_deduct_recurs l e (LENGTH l - 1) (LENGTH l - 1) LENGTH e)
```

The data type of function zsyn_deduct is (α *list list* $\rightarrow$ (α *list* $\#$ α *list list*)*list* $\rightarrow$ α *list list*). It accepts the initial list of molecules and the list of valid EVFs and returns a list of final outcomes of the experiment under the given conditions. Next, the output of the function zsyn_deduct has to be checked if the desired molecule is present in the list. This can be done by using the elimination of the Z-Conjunction rule given in Definition 4.1.

The formal definitions, presented in this section, allow us to recursively check all the possible combinations of the initial molecules against the first elements of given EVFs. In case of a match, the corresponding EVF is applied by replacing the reacting molecules with their outcome in the molecule list and the process restarts again to find other possible matches from the new list of molecules. This process terminates when no more molecules are found to be reacting with each other and at this point we will have the list of post-reaction molecules. The desired result can then be obtained from these molecules using the elimination of Z-Conjunction rule, given in Definition 4.1. The main benefit of the development, presented in this section, is that it facilitates automated reasoning about the molecular biological experiments within the sound core of a theorem prover.

4.1 Formal Verification of Zsyntax Properties

In order to ensure the correctness and soundness of our definitions, we use them to verify a couple of properties representing the most important characteristics of molecular reactions. The first property deals with the case when there is no combination of reacting molecules in the list of molecules and in this case we verify that after the Zsyntax based experiment execution both the pre and post-experiment lists of molecules are the same. The second property captures the behavior of the scenario when the given list of molecules contains only one set of reacting molecules and in this case we verify that after the Zsyntax based experiment execution the post-experiment list of molecules contains the products of the reacting molecules minus its reactants along with the remaining molecules provided initially. We represent these scenarios as formally specified properties in higher-order logic using our formal definitions, given in the previous section. These properties are then formally verified in higher-order logic theorem prover.

4.1.1 Scenario 1: No Reaction

We verify the following theorem for the first scenario:

Theorem 4.1. Non-Reacting Molecules

```
⊢ ∀ e l.
        A1: ~(NULL e) ∧ A2: ~(NULL l) ∧
        A3: (∀ a x y. MEM a e ∧ x < LENGTH l ∧ y < LENGTH l
             ⇒ ~MEM (FST a) [HD (zsyn_conjun_intro l x y)])
     ⇒ zsyn_deduct l e = l
```

The variables `e` and `l` represent the lists of EVFs and molecules, respectively. The first two assumptions (A1-A2) ensure that both of these lists have to be non-empty, which are the pre-conditions for a molecular reaction to take place. The next assumption (A3) in the assumption list of Theorem 4.1 represents the formalization of the no-reaction-possibility condition as according to this condition no first element of any pair in the list of EVFs `e` is a member of the head of the list formed by the function `zsyn_conjun_intro`, which picks the elements corresponding to the two given indices (that range over the complete length of the list of molecules `l`) and appends them as a flattened single element on the given list `l`. This constraint is quantified for all variables `a`, `x` and `y` and thus ensures that no combination of molecules in the list `l` matches any one of the first elements of the EVF list `e`. Thus, under this constraint, no reaction can take place for the given lists `l` and `e`. The conclusion of Theorem 4.1 represents the scenario that the output of our formalization of Zsyntax based reaction would not make any change in the given molecule list `L` and thus verifies that under the no-reaction-possibility condition our formalization also did not update the molecule list.

The verification of this theorem is interactively done by ensuring the no-update scenario for all molecule manipulation functions, i.e., `zsyn_EVF`, `zsyn_recurs1`, `zsyn_recurs2` and `zsyn_deduct_recurs`, under the no-reaction-possibility condition [25]. For example, the corresponding theorem for `zsyn_EVF` function is as follows:

Theorem 4.2. No Update for List of EVFs

```
⊢ ∀ e l x y p.
        A1: ~(NULL e) ∧ A2: ~(NULL l) ∧ A3: x < LENGTH l ∧ A4: y < LENGTH l ∧
        A5: p < LENGTH e ∧ A6: (∀ a. MEM a e ⇒ ~MEM (FST a) [HD l])
     ⇒ zsyn_EVF l e p x y = (F,TL l)
```

The assumptions (A1-A5) of above theorem ensure that both lists `l` and `e` are not empty and the arguments of the function `zsyn_EVF` are bounded by the `LENGTH` of `l` and `e`. The last assumption (A6) in the assumption list models the no-reaction-possibility condition in the context of the function `zsyn_EVF`. The conclusion of the theorem states that no update takes place under the given conditions by ensuring that the function `zsyn_EVF` returns a pair with the first element being `F` (False), representing no match, and the second element being equal to `TL l`, which is actually equal to the original list `l` since an element was appended on head of `l` by the parent function.

4.1.2 Scenario 2: Single Reaction

The second scenario complements the first scenario and caters for the case when a reaction is possible and we verify that the molecules list is indeed updated based on the outcomes of that reaction. In order to be able to track the reaction and the corresponding update, we limit ourselves to only one reaction in this scenario but since

we verify a generic theorem (universally quantified) for all possibilities our result can be extended to cater for multiple reactions as well. The theorem corresponding to this scenario 2 is as follows:

Theorem 4.3. Molecule List Update in Case of a Reaction

```
⊢ ∀ e l z x' y'.
    A1: ~ NULL e ∧ A2: ~ NULL (SND (EL z e)) ∧ A3: 1 < LENGTH l ∧
    A4: x' ≠ y' ∧ A5: x' < LENGTH l ∧ A6: y' < LENGTH l ∧ A7: z < LENGTH e ∧
    A8: ALL_DISTINCT (APPEND L (SND (EL z e))) ∧
    A9: (∀ a b. a ≠ b ⇒ FST (EL a e) ≠ FST (EL b e)) ∧
    A10: (∀ k x y. x < LENGTH k ∧ y < LENGTH k ∧
   (∀ j. MEM j k ⇒ MEM j l ∨ ∃ q. MEM q e ∧ MEM j (SND q)) ⇒
     if (EL x k = EL x' l) ∧ (EL y k = EL y' l)
       then HD (zsyn_conjun_intro k x y) = FST (EL z e)
       else ∀ a. MEM a e ⇒ FST a ≠ HD (zsyn_conjun_intro k x y))
   ⇒ zsyn_deduct l e = zsyn_delet (APPEND l (SND (EL z e))) x' y'
```

The first two assumptions (A1-A2) ensure that neither the list `e`, i.e., the list of EVFs, nor the second element of the pair at index `z` of the list `e` is empty. Similarly, the third assumption (A3) ensures that the list `l`, i.e., the list of initial molecules, contains at least two elements. These constraints ensure that we can have at least one reaction with the resultant being available at index `z` of the EVF list. The next four assumptions (A4-A7) ensure that the indices `x'` and `y'` are distinct and these along with the index `z` fall within the range of elements of their respective lists of molecules `l` or EVFs `e`. According to the next assumption (A8), i.e., `ALL DISTINCT (APPEND l (SND (EL z e)))`, all elements of the list `l` and the resulting molecules of the EVF at index `z` are distinct, i.e., no molecule can be found two or more times in the initial list `l` or the post-reaction list `e`. The next assumption (A9), i.e., `(∀ a b. a ≠ b ⇒ FST (EL a e) ≠ FST (EL b e))`, guarantees that all first elements of the pairs in list `e` are also distinct. Note that this is different from the previous condition since the list `e` contains pairs as elements and the uniqueness of the pairs does not ensure the uniqueness of its first elements. The final condition (A10) models the scenario where there is only one pair of reactants present in the reaction. According to the assumptions of this implication condition, the variable `k` is used to represent a list that only has elements from list `l` or the second elements of the pairs in list `e`. Thus, it models the molecules list in a live experiment. Moreover, the variables `x` and `y` represent the indices of the list `k` and thus they must have a value less than the total elements in the list `k` (since the first element is indexed `0` in the HOL Light formalization of lists). Now, if the indices `x` and `y` become equal to `x'` and `y'`, respectively, then the head element of the `zsyn_conjun_intro k x y` would be equal to `FST` of `EL z e`. Otherwise, for all other values of indices `x` and `y`, no combination of molecules obtained by `HD(zsyn_conjun_intro k x y)` would be equal to the first element of any pair of the list `e`. Thus, the if case ensures that the variables `x'` and `y'` point to the reacting molecules in the list of molecules `l` and the variable `z` points to their corresponding resultant molecule in the EVF list. Moreover, the else case ensures that there is only one set of reacting molecules in the list `l`. The conclusion of the theorem formally describes the scenario when the resulting element, available at the location `z` of the EVF list, is appended to the list of molecules while the elements available at the indices `x'` and `y'` of `l` are removed during the execution of the function `zsyn_deduct` on the given lists `l` and `e`.

The proof of Theorem 4.3 is again based on verifying sub-goals corresponding to this scenario for all the sub-functions, i.e., `zsyn_EVF`, `zsyn_recurs1`, `zsyn_recurs2` and `zsyn_deduct_recurs`. The formal reasoning for all of these proofs involved various properties of the `delet` function for a list element and some of the key theorems developed for this purpose in our development are given in Table 3.

The formalization described in this section consumed about 500 man hours and approximately 2000 lines of code, mainly due to the undecidable nature of higher-order logic. However, this effort raises the confidence level on the correctness of our formalization of Zsyntax . This fact distinguishes our work from all the other formal methods based techniques used in the context of BRNs, where the deduction rules are applied without being formally checked. Moreover, our formally verified theorems can also be used in the formal analysis of molecular pathways. As the assumptions of these theorems provide very useful insights about the constraints under which a reaction or no reaction would take place.

5 Formalization of Reaction Kinetics

In this section, we present the higher-order-logic formalization of the reaction kinetics described in Section 3.

Table 3: Formally Verified Properties of the `delet` Function

Signature	Theorem
`delet_ASSOC_THM`	$\vdash \forall$ `l e x. x` $<$ `LENGTH l` $\Rightarrow$ `delet (APPEND l e) x` $=$ `APPEND (delet l x) e`
`delet_LENGTH_THM`	$\vdash \forall$ `l e x. x` $<$ `LENGTH l` $\Rightarrow$ `LENGTH (delet l x)` $=$ `LENGTH l` $-$ `1`
`delet_EL_THM`	$\vdash \forall$ `l x y. x` $<$ `y` $\wedge$ `y` $<$ `LENGTH l` $\wedge$ `1` $<$ `LENGTH l` $\Rightarrow$ `EL x l` $=$ `EL x (delet l y)`
`delet_DISTINCT_THM`	$\vdash \forall$ `l y. y` $<$ `LENGTH l` $\wedge$ `ALL_DISTINCT l` $\Rightarrow$ `ALL_DISTINCT (delet l y)`
`delet_MEM_THM`	$\vdash \forall$ `l a x. x` $<$ `LENGTH l` $\wedge$ `MEM a (delet l x)` $\Rightarrow$ `MEM a l`
`delet_NOT_MEM_THM`	$\vdash \forall$ `l x. ALL_DISTINCT l` $\wedge$ `x` $<$ `LENGTH l` $\Rightarrow\sim$ `MEM (EL x l) (delet l x)`

5.1 Generalized Model Formulation

In reaction kinetic based modeling, a biological pathway or a network is represented by a set of biochemical reactions, which can be classified as reversible and irreversible. We can formally define this fact by an inductive enumerating data-type in HOL Light as follows:

Definition 5.1. Reaction Type
```
define_type "reaction_type = irreversible | reversible"
```

A general model of the biological reaction consists of a list of reactants, a list of products and corresponding kinetic rate constants (forward and reverse). We model a biological entity as a pair ($\mathbb{N}$, $\mathbb{R}$), where the first element represents the stoichiometry and second element is the concentration of the molecule. For example, $2A$ in a reaction can be represented as (2,A). We formally model a biological reaction as the following type abbreviation:

Definition 5.2. Biological Reaction
```
new_type_abbrev "bio_reaction",
               :(reaction_type × ((num × real)list × (num × real)list × (real × real)))
```

In the above definition, a biological reaction is a pair whose first element represents type of the reaction `reaction_type` (Definition 5.1) and the second element is itself a 3-tuple. The first element (`num` $\times$ `real`)`list` of the 3-tuple is a list of the reactants represented in the form of a pair in which the first element of the pair represents the stoichiometry and second element is the concentration of a single reactant. Similarly, the second element (`num` $\times$ `real`)`list` of the 3-tuple is a list of the products of the reaction represented in the same format as reactants. Finally, the third element of the 3-tuple is also a pair (`real` $\times$ `real`), which represents the kinetic rate constants of the reaction. In the case of irreversible reaction, the first element of this pair is the kinetic rate constant of the reaction and second element is zero. Whereas, in the case of the reversible reaction, the first element is the forward kinetic rate constant and the second element represents the reverse kinetic rate constant of the reaction.

In order to obtain an ODE model from a reaction based model of a biological system, we need to find out the flux vector and the stoichiometric matrix. We first formalize the flux vector of the overall reaction which can be computed by the law of mass action as described in Section 3. This requires the concentration of each molecule

to be transformed in the exponent form where the concentration of a molecule is represented as the base and the corresponding stoichiometry as the exponent. We define the following functions to perform this task.

Definition 5.3. Product of the Concentrations

```
⊢ ∀ h t.  flux_irr [ ] = &1 ∧
          flux_irr (CONS h t) =
             if FST h = 0
                then flux_irr t
                else SND h pow FST h * flux_irr t
```

The function flux_irr accepts a list of reactants in the form of pair and returns a real number which is the product of the concentration raised the stoichiometry of all the reactants in the reaction.

The flux of an irreversible reaction is given by the following definition below:

Definition 5.4. Flux of an Irreversible Reaction

```
⊢ ∀ products_list rate reactants_list.
          gen_flux_irreversible reactants_list products_list rate =
                rate * flux_irr reactants_list
```

The function gen_flux_irreversible has the type $((num \times real)\ list \rightarrow (num \times real)\ list \rightarrow\ real \rightarrow\ real)$ and takes a list of reactants, a list of products and the first element of the kinetic rate constant pair and returns the flux of an irreversible reaction.

The function for the flux of reversible reaction (Equation 6) is given as follows:

Definition 5.5. Flux of a Reversible Reaction

```
⊢ ∀ rate_1 reactants_list rate_2 products_list.
          gen_flux_reversible reactants_list products_list rate_1 rate_2 =
                rate_1 * flux_irr reactants_list - rate_2 * flux_irr products_list
```

Next, we combine the above definitions into one uniform definition as follows:

Definition 5.6. Flux of a Single Reaction

```
⊢ ∀ t R P k1 k2.
          flux_sing (t,R,P,k1,k2) =
             if t = irreversible
                then gen_flux_irreversible R P k1
                else gen_flux_reversible R P k1 k2
```

The type of the function flux_sing is (*bio_reaction* → *real*). It takes an element of data type (*bio_reaction*) and output the value of flux of that reaction. In this function, there is a condition on the type of the reaction, i.e., if it is irreversible, then this function will return the flux of irreversible reaction, otherwise it will return the flux of the reversible reaction.

A general biological pathway has a chain of reversible and irreversible reactions in any arbitrary sequence. We can obtain the flux vector for a chain of reactions of biological system by the following HOL Light function:

Definition 5.7. Flux Vector

```
⊢ ∀ M. flux M = vector (MAP flux_sing M)
```

The function flux accepts a list of reactions (*bio_reaction list*) and returns a vector ($\mathbb{R}^n$) containing the fluxes of all the reactions, where n is the length of the list M. Here, the HOL Light function MAP is used to map the function flux_sing on every element of the list M.

Our next step is to formalize the notion of stoichiometric matrix which indeed is a collection of column vectors. Each column of the stoichiometric matrix can be defined as follows:

Definition 5.8. Column of the Stoichiometric Matrix

```
⊢ ∀ h t h2 h1 t1 t2.
   stioch_mat_column [ ] [ ] = [ ] ∧
   stioch_mat_column (CONS h t) [ ] = [ ] ∧
   stioch_mat_column [ ] (CONS h t) = [ ] ∧
   stioch_mat_column (CONS h1 t1) (CONS h2 t2) =
     CONS (&(FST h2) - &(FST h1)) (stioch_mat_column t1 t2)
```

The above function has a data type of $((num \times real)\ list \rightarrow (num \times real)\ list \rightarrow\ real\ list)$. The function `stioch_mat_column` accepts a list of the reactants and a list of products and returns a list containing the corresponding column of the stoichiometric matrix. In order to obtain a single entry, the stoichiometry of the product is subtracted from the stoichiometry of the reactant.

Definition 5.9. Vector of the Stoichiometric Matrix Column

```
⊢ ∀ t k1 k2 R P. st_matrix_sing (t,R,P,k1,k2) = vector (stioch_mat_column R P)
```

The function `st_matrix_sing` takes a biological reaction (*bio_reaction*) and returns a vector ($\mathbb{R}^m$), which corresponds to the column of the stoichiometric matrix.

Finally, we can obtain the stoichiometric matrix (in transposed form) by applying the above function to the list of biological reactions, as defined in the following definition:

Definition 5.10. Stoichiometric Matrix

```
⊢ ∀ M. st_matrix M = vector (MAP st_matrix_sing M)
```

The definition `st_matrix` takes a list of biological reaction (`bio_reaction`) and returns a matrix having `n` rows and `m` columns, where `n` and `m` are the number of reactions and number of the species, respectively. The function `MAP` applies the definition `st_matrix_sing` on every element of the list `M`. Further, the stoichiometric matrix can be obtained by taking the transpose of the function `st_matrix`.

Next, we define a helper function that maps a real derivative over each function of the list.

Definition 5.11. Derivative of a List of Functions

```
⊢ ∀ h t x.
     map_real_deriv [ ] x = [ ] ∧
     map_real_deriv (CONS h t) x = APPEND [real_derivative h x] (map_real_deriv t x)
```

The above function accepts a list (`real_list`) containing the concentrations of all the species taking part in the reaction. It uses a HOL Light function `real_derivative`, which represents the real-valued derivative of a function and maps it over the each element of the list (`real_list`). We use `map_real_deriv` to define a function which takes a list of the concentrations of the species and returns a vector with each element represented in the form of a real-valued derivative.

Definition 5.12. Derivative of a Vector

```
⊢ ∀ L t.  entities_deriv_vec L t = vector (map_real_deriv L t)
```

We can utilize this infrastructure to model arbitrary biological networks consisting of any number of reactions. For example, a biological network consisting of list of `E` biological species and `M` biological reactions can be represented by the following general kinetic model:

```
((entities_deriv_vec E t):real^m) = transp((st_matrix M):real^m^n) ** flux M
```

5.2 Properties of Reaction Kinetics Based Modeling

We used the formalization of the reaction kinetics to verify the generic models of biological reactions, such as irreversible consecutive reactions, reversible and irreversible mixed reactions. The main ideal is to express

the given biological network as a kinetic model and verify that the given solution (mathematical expression) of each biological entity satisfies the resulting set of coupled differential equations. This verification is quite important as such expressions are used to predict the outcomes of various drugs and understand the evolution of different molecules. In the following, we present some commonly used reaction schemes, their formal modeling and verification process in HOL Light.

5.2.1 The Irreversible Consecutive Reactions

We consider a general irreversible consecutive reaction scheme as shown in Figure 2. This configuration represents two irreversible reactions. In the first reaction, `A` is the reactant and `B` is the product whereas k_1 represents the kinetic rate constant of the reaction. Similarly, in the second reaction, `B` is the reactant, `C` is the product and k_2 is its kinetic rate constant. We formally model this reaction scheme in HOL Light as follows:

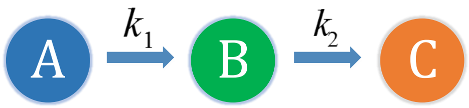

Figure 2: The Irreversible Consecutive Reactions

Definition 5.13. The Irreversible Consecutive Reaction

```
⊢ ∀ k1 A B C t k2.
  rea_sch_01 A B C k1 k2 t =
  [irreversible,[1,A t; 0,B t; 0,C t],[0,A t; 1,B t; 0,C t],k1,&0;
   irreversible,[0,A t; 1,B t; 0,C t],[0,A t; 0,B t; 1,C t],k2,&0]
```

where the function `rea_sch_01` accepts the concentrations of the species `A`, `B`, `C`, the kinetic rate constants `k1`, `k2`, a real-valued time variable `t` and returns a list of two irreversible biological reactions (*bio_reaction*).

Our next step is to verify the solution of the kinetic model of the above reaction scheme:

Theorem 5.1. Solution of the ODE model of Reaction Scheme 01

```
⊢ ∀ A B C t k1 k2.
  A1: &0 < k1 ∧ A2: &0 < k2 ∧ A3: ∼(k2 - k1 = &0) ∧
  A4: A (&0) = A0 ∧ A5: B (&0) = &0 ∧ A6: C (&0) = &0 ∧
  A7: (∀ t.  A t = A0 ∗ exp (--k1 ∗ t)) ∧
  A8: (∀ t.  B t = A0 ∗ k1 / (k2 - k1) ∗ (exp (--k1 ∗ t) - exp (--k2 ∗ t))) ∧
  A9: (∀ t.  C t = A0 ∗ (&1 - k2 / (k2 - k1) ∗ exp (--k1 ∗ t) - k1 / (k1 - k2)
             ∗ exp (--k2 ∗ t)))
  ⇒ entities_deriv_vec [A; B; C] t =
     transp (st_matrix (rea_sch_01 A B C k1 k2 t)) ∗∗
     flux (rea_sch_01 A B C k1 k2 t)
```

where ∗∗ represents the matrix-vector multiplication. The HOL Light function `transp` accepts a matrix of any order and returns the correspnding transpose of that matrix. The first two assumptions (A1-A2) ensure that the kinetic rate constants of all the reactions are non-negative. The assumption (A3), i.e., `∼(k2 - k1 = &0)`, guarantees that the denominators of the expressions of `B t` and `C t` are not zero in order to avoid singularities. The next three conditions (A4-A6) are the initial concentrations of the species. Finally, the last three assumptions (A7-A9) are the concentrations of the species `A`, `B` and `C` at any time `t`. These are the solutions of the ODE model. The conclusion of the theorem describes the kinetic model for the given reaction.

We start the proof process by rewriting the left-hand-side using the following lemma which results in a vector containing the derivatives of all the species.

Lemma 5.1. Entities Derivative Vector

```
⊢ ∀ A B C k1 k2 t.
      entities_deriv_vec [A; B; C] t =
        vector [real_derivative A t; real_derivative B t; real_derivative C t]
```

The above lemma can easily be proved by using a simplifier KINETIC_SIMP, which we developed by using the rewriting and inference rules of HOL Light to automate some parts of the formal reasoning process.

Next, we need to compute the stoichiometric matrix on the right-hand-side of Theorem 5.1, which we derive in the following lemma.

Lemma 5.2. Stoichiometric Matrix

```
⊢ ∀ A B C k1 k2 t.
  transp (st_matrix (rea_sch_01 A B C k1 k2 t)) =
  vector [vector [&0 - &1; &0 - &0]; vector [&1 - &0; &0 - &1]; vector [&0 - &0; &1 - &0]]
```

The left-hand-side of the Lemma 5.2 consists of the function `st_matrix`, which accepts a list `rea_sch_01` of the biological reactions and returns the transpose of the stoichiometric matrix. We prove this theorem automatically by KINETIC_SIMP.

Next, we derive the flux vector from the function `flux (rea_sch_01 A B C k1 k2 t)` on the right-hand-side of the conclusion of the Theorem 5.1, as follows:

Lemma 5.3. Flux Vector

```
⊢ ∀ A B C k1 k2 t.
  flux (rea_sch_01 A B C k1 k2 t) = vector [k1 * A t; k2 * B t]
```

At this point, we multiply the stoichiometric matrix with the flux vector, along with some arithmetic reasoning, to obtain three differential equations. We discharge this last subgoal with the help of following important lemma:

Lemma 5.4. Solution of the ODEs

```
⊢ ∀ A B C t k1 k2.
  A1: &0 < k1 ∧ A2: &0 < k2 ∧ A3: ~(k2 - k1 = &0) ∧
  A4: A (&0) = A0 ∧ A5: B (&0) = &0 ∧ A6: C (&0) = &0 ∧
  A7: (∀ t. A t = A0 * exp (--k1 * t)) ∧
  A8: (∀ t. B t = A0 * k1 / (k2 - k1) * (exp (--k1 * t) - exp (--k2 * t))) ∧
  A9: (∀ t. C t = A0 * (&1 - k2 / (k2 - k1) * exp (--k1 * t) - k1 / (k1 - k2)
            * exp (--k2 * t)))
  ⇒ real_derivative (λ t. A t) t = --k1 * A t ∧
    real_derivative (λ t. B t) t = k1 * A t - k2 * B t ∧
    real_derivative (λ t. C t) t = k2 * B t
```

The proof of this lemma is based on some properties of the real-valued derivatives available in the HOL Light library along with some real analysis. We also utilized DIFF_TAC, which is a very powerful tactic and helps to automatically prove the real-valued derivatives of arbitrary and difficult functions.

5.2.2 The Consecutive Reactions with the Second Step being Reversible

The second reaction scheme consists of the consecutive reactions with the second step being reversible as shown in Figure 3. In the irreversible reaction, A and B are the reactant and product, respectively, whereas k_1 is the kinetic rate constant of the reaction. Since any reversible reaction can be written as two irreversible reactions, so the first irreversible reaction has B, C and the forward kinetic reaction constant k_2 as the reactant, product and the kinetic rate constant, respectively. Similarly, the parameters C, B and k_3 are the reactant, product and kinetic rate constant of the second irreversible reaction, respectively. We formally model this scheme as follows:

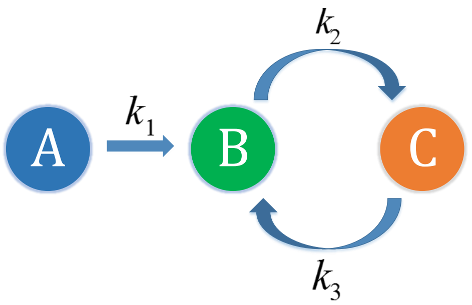

Figure 3: The Consecutive Reactions with the Second Step being Reversible

Definition 5.14. The Consecutive Reactions with the Second Step Being Reversible

```
⊢ ∀ k1 A B C t k2 k3.
  rea_sch_02 A B C k1 k2 k3 t =
  [irreversible,[1,A t; 0,B t; 0,C t],[0,A t; 1,B t; 0,C t],k1,&0;
   reversible,[0,A t; 1,B t; 0,C t],[0,A t; 0,B t; 1,C t],k2,k3]
```

where the function `rea_sch_02` accepts the concentrations of the species `A`, `B`, `C`, the kinetic rate constants `k1, k2, k3`, a real-valued time variable `t` and returns the list of biological reactions (*bio_reaction*).

We verify the solution of the ODE model using the following theorem:

Theorem 5.2. Solution of the ODE model of Reaction Scheme 02

```
⊢ ∀ A B C t k1 k2 k3.
  A1:  &0 < k1 ∧ A2:  &0 < k2 ∧ A3:  &0 < k3 ∧
  A4:  A (&0) = A0 ∧ A5:  B (&0) = &0 ∧ A6:  C (&0) = &0 ∧
  A7:  r1 = k1 ∧ A8:  r2 = k2 + k3 ∧ A9:  ∼(r1 = r2) ∧
  A10:  (∀ t.  A t = A0 ∗ exp (--k1 ∗ t)) ∧
  A11:  (∀ t.  B t = k1 ∗ A0 ∗ (k3 / (r1 ∗ r2) + (r2 - k3) / (r2 ∗ (r1 - r2)) ∗ exp (--r2 ∗ t)
               + (k3 - r1) / (r1 ∗ (r1 - r2)) ∗ exp (--r1 ∗ t))) ∧
  A12:  (∀ t.  C t = k1 ∗ k2 ∗ A0 ∗ (&1 / (r1 ∗ r2) + &1 / (r1 ∗ (r1 - r2)) ∗ exp (--r1 ∗ t)
               - &1 / (r2 ∗ (r1 - r2)) ∗ exp (--r2 ∗ t)))
  ⇒ entities_deriv_vec [A; B; C] t =
     transp (st_matrix (rea_sch_02 A B C k1 k2 k3 t)) **
     flux (rea_sch_02 A B C k1 k2 k3 t)
```

The first three assumptions (A1-A3) of Theorem 5.2 guarantees the non-negativity of the kinetic rate constants of the reactions. The next three assumptions (A4-A6) are the initial concentrations of the species participating in the reaction. The assumptions (A7) and (A8) are introduced to simplify the expressions for the concentrations of the species. The next assumption (A9) jointly with the first three assumptions (A1-A3) ensures that the denominators of the expressions for `B t` and `C t` are not zero in order to avoid singularities. The last three assumptions (A10-A12) are the concentrations of the species `A`, `B` and `C` at any time `t`. These are the solutions of the system of ODEs modeling the biological reactions. The conclusion of the theorem presents the ODE model for the given reaction scheme which is a vector equation. The verification process of this theorem is similar to the one of Theorem 5.1, and more details can be found in the source code of our formalization [36].

5.2.3 The Consecutive Reactions with First Step as a Reversible Reaction

In this scheme, the first reaction is reversible and the second reaction is irreversible as shown in Figure 4. The reversible reaction can be equivalently written as two irreversible reactions with k_1 and k_2 as their kinetic rate constants. In the first irreversible reaction, `A` and `B` are the reactant and product, respectively, whereas in the second reaction, `B` and `A` are the reactant and product, respectively. For the second step, `B`, `C` and k_3 are the reactant, product and kinetic rate constant, respectively. The corresponding formal definition is given as follows:

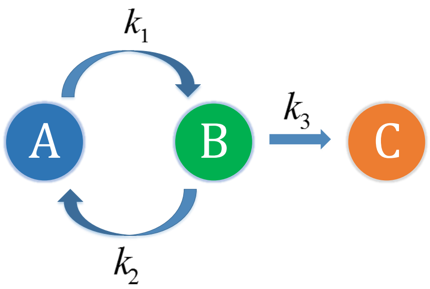

Figure 4: The Consecutive Reactions with First Step as a Reversible Reaction

Definition 5.15. The Consecutive Reactions with First Step as a Reversible Reaction

```
⊢ ∀ k1 k2 A B C t k3.
  rea_sch_03 A B C k1 k2 k3 t =
  [reversible,[1,A t; 0,B t; 0,C t],[0,A t; 1,B t; 0,C t],k1,k2;
   irreversible,[0,A t; 1,B t; 0,C t],[0,A t; 0,B t; 1,C t],k3,&0]
```

The function `rea_sch_03` takes the concentrations of the species `A`, `B`, `C`, the kinetic rate constants `k1, k2, k3` and the time variable `t` and returns the corresponding list of biological reactions (*bio_reaction*).
The solution of the ODE model of this reaction scheme is given by the following theorem:

Theorem 5.3. Solution of the ODE model of Reaction Scheme 03

```
⊢ ∀ A B C t k1 k2 k3.
  A1: &0 < k1 ∧ A2: &0 < k2 ∧ A3: &0 < k3 ∧
  A4: A (&0) = A0 ∧ A5: B (&0) = &0 ∧ A6: C (&0) = &0 ∧
  A7: r1 * r2 = k1 * k3 ∧ A8: r1 + r2 = k1 + k2 + k3 ∧
  A9: ~(r1 = &0) ∧ A10: ~(r2 = &0) ∧ A11: ~(r1 = r2) ∧
  A12: (∀ t. A t = A0 / (r2 - r1) * ((k2 + k3 - r1) * exp (--r1 * t) - (k2 + k3 - r2)
             * exp (--r2 * t))) ∧
  A13: (∀ t. B t = (A0 * k1) / (r2 - r1) * (exp (--r1 * t) - exp (--r2 * t))) ∧
  A14: (∀ t. C t = A0 * (&1 + (k1 * k3) / (r1 * (r1 - r2)) * exp (--r1 * t)
             + (k1 * k3) / (r2 * (r2 - r1)) * exp (--r2 * t)))
  ⇒ entities_deriv_vec [A; B; C] t =
     transp (st_matrix (rea_sch_03 A B C k1 k2 k3 t)) **
     flux (rea_sch_03 A B C k1 k2 k3 t)
```

The first three assumptions (A1-A3) of the above theorem ensure that the kinetic rate constants of the reactions are non-negative. The next three assumptions (A4-A6) are the initial concentrations of the species participating in the reaction. The assumptions (A7) and (A8) are introduced to simplify the expressions for the concentrations of the species. The next three assumptions (A9-A11) guarantee that the denominators of the expressions for `A t`, `B t` and `C t` are non-zero. The last three assumptions (A12-A14) are the concentrations of the species `A`, `B` and `C` at time `t`, representing the solutions of the system of the corresponding ODEs. We verify this theorem using the same steps used for the verification of Theorems 5.1 and 5.2.

5.2.4 The Consecutive Reactions with a Reversible Step

In this reaction scheme, we consider the consecutive reactions with one reversible and one irreversible reaction step as shown in Figure 5. We formalize this two step biological reaction scheme as follows:

Definition 5.16. The Consecutive Reactions with a Reversible Step

```
⊢ ∀ k1 k2 A B C t k3.
rea_sch_04 A B C k1 k2 k3 t =
[reversible,[1,A t; 0,B t; 0,C t],[0,A t; 1,B t; 0,C t],k1,k2;
 irreversible,[1,A t; 0,B t; 0,C t],[0,A t; 0,B t; 1,C t],k3,&0]
```

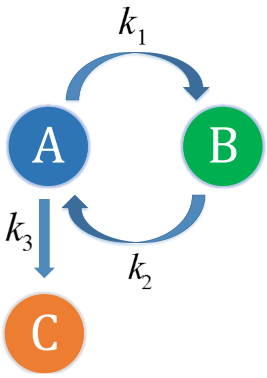

Figure 5: The Consecutive Reactions with a Reversible Step

The above definition accepts the concentrations of the species `A`, `B`, `C`, the kinetic rate constants `k1, k2, k3`, the time variable `t` and returns the list of corresponding biological reactions (*bio_reaction*).

We verify the solution of the ODE model of this reaction as follows:

Theorem 5.4. Solution of the ODE model of Reaction Scheme 04

```
⊢ ∀ A B C t k1 k2 k3.
  A1: &0 < k1 ∧ A2: &0 < k2 ∧ A3: &0 < k3 ∧
  A4: A (&0) = A0 ∧ A5: B (&0) = &0 ∧ A6: C (&0) = &0 ∧
  A7: r1 * r2 = k2 * k3 ∧ A8: r1 + r2 = k1 + k2 + k3 ∧
  A9: ~(r1 = &0) ∧ A10: ~(r2 = &0) ∧ A11: ~(r1 = r2) ∧
  A12: (∀ t. A t = A0 * ((k2 - r1) / (r2 - r1) * exp (--r1 * t) - (k2 - r2) / (r2 - r1)
             * exp (--r2 * t))) ∧
  A13: (∀ t. B t = (k1 * A0) / (r2 - r1) * (exp (--r1 * t) - exp (--r2 * t))) ∧
  A14: (∀ t. C t = A0 * (&1 + (k3 * (k2 - r1)) / (r1 * (r1 - r2)) * exp (--r1 * t)
             + (k3 * (k2 - r2)) / (r2 * (r2 - r1)) * exp (--r2 * t))) ∧
  ⇒ entities_deriv_vec [A; B; C] t
    transp (st_matrix (rea_sch_04 A B C k1 k2 k3 t)) **
    flux (rea_sch_04 A B C k1 k2 k3 t)
```

The detailed proof of this theorem can be found in the source code [36] along with the details about required lemmas.

This completes our formal verification of some commonly used reaction schemes. The verification of these solutions requires user interaction but the strength of these theorems lies in the fact that they have been verified for the arbitrary values of parameters, such as k_1 and k_2, etc. This is a unique feature of higher-order-logic theorem proving and is not possible in the case of simulation where such continuous expressions are tested for few samples of such parameters. Another important aspect is the explicit presence of all the assumptions required to verify the set of ODEs. For example, such assumptions for the above-mentioned reaction schemes are not mentioned in [37].

6 Case Studies

In this section, we use our proposed framework to formally reason about three real-world systems biology case studies: First, we formally analyse the reaction involving the phosphorylation of TP53. Second, we formally derive the time evolution expressions of different tumor cell types which are used to predict the tumor population and volume at a given time instant. Third, we take another model for the growth of tumor cells and perform both the Zsyntax and reaction kinetic based formal analysis using our proposed formalizations presented in the Sections 4 and 5 of the report.

6.1 TP53 Phosphorylation

TP53 gene encodes p53 protein, which plays a crucial role in regulating the cell cycle of multicellular organisms and works as a tumour suppressor for preventing cancer [14]. In this section, we show how this pathway involving TP53 Phosphorylation can be formally verified in HOL Light using our formalization of Zsyntax .

The theorem representing the reaction of the pathway leading from TP53 to p(TP53) [14] can be described in classical Zsyntax format as follows:

$$\texttt{TP53 \& ATP \& Kinase} \vdash \texttt{p(TP53)}$$

Using our formalization, this theorem can be defined in HOL Light as follows:

Theorem 6.1. The reaction of the pathway leading from TP53 to p(TP53)

```
⊢ DISTINCT [TP53; ATP; Kinase; ADP; pTP53] ⟹
    (zsyn_conjun_elimin (zsyn_deduct [[TP53]; [ATP]; [Kinase]]
        [[Kinase; ATP], [[ATP; Kinase]];
         [ATP; Kinase; TP53], [[Kinase]; [pTP53]; [ADP]]]) [pTP53] = [[pTP53]]
```

The first list argument of the function `zsyn_deduct` is the initial aggregate (IA) of molecules that are available for reaction and the second list argument of the function `zsyn_deduct` represents the valid EVFs for this reaction. The EVFs mentioned in the form of pairs and involving the molecules (ATP, Kinase, etc.) are obtained from wet lab experiments, as reported in [4]. The `DISTINCT` function used above makes sure that all molecule variables (from initial aggregate and EVFs) used in this theorem represent distinct molecules. Thus, the function `zsyn_deduct` would deduce the final list of molecules under these particular conditions. The function `zsyn_conjun_elimin` will return the molecule `pTP53` if it is present in the post-reaction list of molecules, as previously described.

Figure 6 shows the pathway leading to p(TP53) in a step-wise manner. The blue-coloured circles show the chemical interactions and green colour represents the desired product in the pathway, whereas each rectangle shows total number of molecules in the reaction at a given time. It is obvious from the figure that whenever a reaction yields a product, the reactants get consumed (no longer remain in the list) hence satisfying the stoichiometry of a reaction.

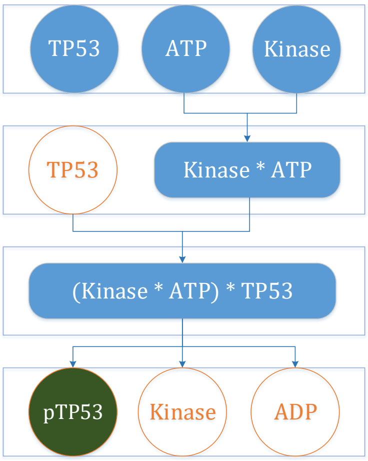

Figure 6: Reaction Representing the TP53 Phosphorylation

As part of this work, we also developed a simplifier `Z_SYNTAX_SIMP` [36] that simplifies the proof with a single iteration of the function `zsyn_deduct_recurs` and works very efficiently with the proofs involving our functions. The proof steps can be completely automated and the proof can be done in one step as well. However, we have kept the reasoning process manual purposefully as this way users can observe the status of the reaction at every iteration, which is a very useful feature to get an insight of what is happening inside a reaction. Each application of `Z_SYNTAX_SIMP` on the reaction, depicted in Figure 6, would result in moving from a state n to $n + 1$.

Our HOL Light proof script is available for download [36], and thus can be used for further developments and analysis of different molecular pathways. It is important to note that formalizing Zsyntax and then verifying its properties was a very tedious effort. However, it took only 6 lines of code to define and verify the theorem related to the above case study in HOL Light, which clearly illustrates the usefulness of our foundational work.

We have shown that our formalization is capable of modeling molecular reactions using Zsyntax inference rules, i.e., given a set of possible EVFs, our formalism can derive a final aggregate **B** from an initial aggregate **A** automatically. In case of a failure to deduce **B**, the proposed method still provides the biologist with all the intermediate steps so that one can examine the reaction in detail and figure out the possible cause of failure.

6.2 Formal Analysis of Tumor Growth based on Cancer Stem Cells (CSC)

In the literature of biology, there is much evidence which supports the hypothesis that malignant tumors (cancers) are originally initiated by different tumor cells, which have similar physiological characterises as of normal stem cells in human body. This hypothesis is widely known as Cancer Stem Cell (CSC) hypothesis [38] and explains that the cancer cell exhibit the ability to self-renewal and can also produce different types of differentiated cells. The mathematical and computational modeling of cancers can provide an in-depth understanding and the prediction of required parameters to shape the clinical research and experiments. This can result in an efficient planning and therapeutic strategies for the accurate patient prognosis. In this report, we consider a kinetic model of cancer based on the Cancer Stem Cell (CSC) hypothesis, which was recently proposed in [39]. In this model, four types of events are considered: 1) CSC self-renewal; 2) maturation of CSCs into P cells; 3) differentiation to D cells; and 4) death of all cell subtypes. All of these type of reactions are driven by different rate constants as shown in Figure 7.

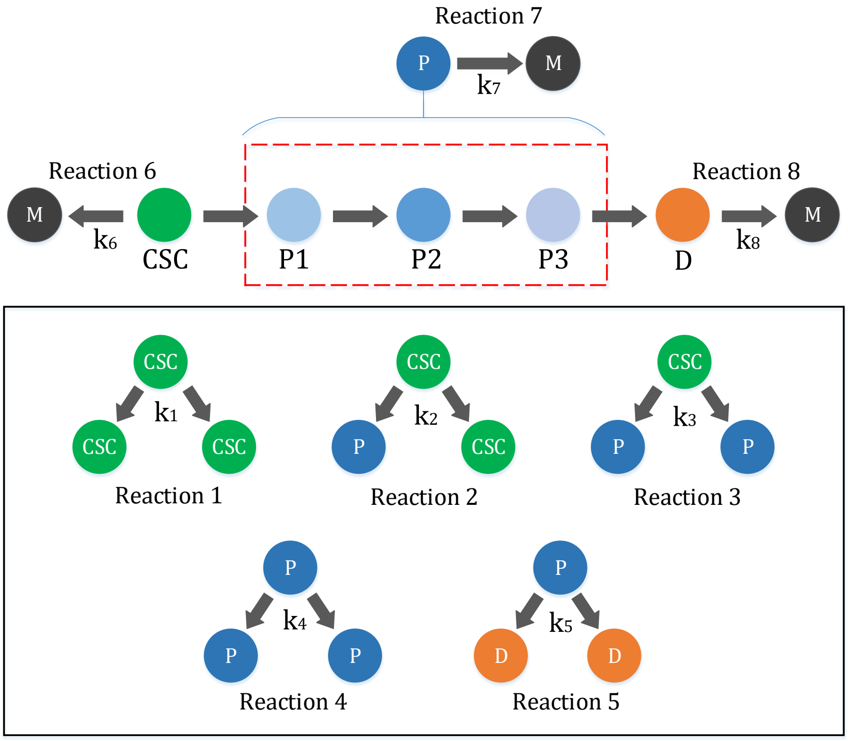

Figure 7: Model for the Tumor Growth [39]

In the following, we provide the possible reactions in the considered model of cancer.

1. Expansion of CSCs can be accomplished through symmetric division, where one CSC can produce two CSCs.

$$\mathrm{CSC} \xrightarrow{k_1} 2\,\mathrm{CSC}$$

2. A CSC can undergo asymmetric division (whereby one CSC gives rise to another CSC and a more differentiated progenitor (P) cell). This P cell possesses intermediate properties between CSCs and differentiated (D) cells.

$$\mathrm{CSC} \xrightarrow{k_2} \mathrm{CSC} + \mathrm{P}$$

3. The CSCs can also differentiate to P cells by symmetric division.

$$\mathrm{CSC} \xrightarrow{k_3} 2\,\mathrm{P}$$

4. The P cells can either self-renew, with a decreased capacity compared to CSCs, or they can differentiate to D cells.

$$\mathrm{P} \xrightarrow{\mathrm{k_4}} 2\,\mathrm{P}$$

$$\mathrm{P} \xrightarrow{\mathrm{k_5}} 2\,\mathrm{D}$$

5. All cellular subtypes can undergo cell death.

$$\mathrm{CSC} \xrightarrow{\mathrm{k_6}} \mathrm{M}$$

$$\mathrm{P} \xrightarrow{\mathrm{k_7}} \mathrm{M}$$

$$\mathrm{D} \xrightarrow{\mathrm{k_8}} \mathrm{M}$$

In order to reduce the complexity of the resulting model, only three subtypes of cells are considered: CSCs, transit amplifying progenitor cells (P), and terminally differentiated cells (D) as shown in Figure 7. This assumption is consistent with several experimental reports [39]. Our main objective is to derive the mathematical expressions, which characterize the time evolution of CSC, P and D. Concretely, such values of these cells should satisfy the set of differential equations that arise in the kinetic model of the proposed tumor growth. Once the expressions of all cell types are known, the total number of tumor cell (N) in the human body can be computed by the formula given as follows:

$$N(t) = CSC(t) + P(t) + D(t) \tag{7}$$

Furthermore, the tumor volume (V) can be calculated, considering that the effective volume contribution of a spherically shaped cell in a spherical tumor (i.e., $4.18 \times 10^{-6} mm^3/cell$), as follows:

$$V(t) = 4.18 \times 10^{6} N(t) \tag{8}$$

We formally model the tumor growth model in HOL Light as follows:

Definition 6.1. Tumor Growth Model

```
⊢ ∀ CSC P D M k1 k2 k3 k4 k5 k6 k7 k8 t.
  tumor_growth_model CSC P D M k1 k2 k3 k4 k5 k6 k7 k8 t =
   [(irreversible,([(1,CSC t);(0,P t);(0,D t);(0,M t)],
                   [(2,CSC t);(0,P t);(0,D t);(0,M t)],(k1,&0))) ;
    (irreversible,([(1,CSC t);(0,P t);(0,D t);(0,M t)],
                   [(1,CSC t);(1,P t);(0,D t);(0,M t)],(k2,&0))) ;
    (irreversible,([(1,CSC t);(0,P t);(0,D t);(0,M t)],
                   [(0,CSC t);(2,P t);(0,D t);(0,M t)],(k3,&0))) ;
    (irreversible,([(0,CSC t);(1,P t);(0,D t);(0,M t)],
                   [(0,CSC t);(2,P t);(0,D t);(0,M t)],(k4,&0))) ;
    (irreversible,([(0,CSC t);(1,P t);(0,D t);(0,M t)],
                   [(0,CSC t);(0,P t);(2,D t);(0,M t)],(k5,&0))) ;
    (irreversible,([(1,CSC t);(0,P t);(0,D t);(0,M t)],
                   [(0,CSC t);(0,P t);(0,D t);(1,M t)],(k6,&0))) ;
    (irreversible,([(0,CSC t);(1,P t);(0,D t);(0,M t)],
                   [(0,CSC t);(0,P t);(0,D t);(1,M t)],(k7,&0))) ;
    (irreversible,([(0,CSC t);(0,P t);(1,D t);(0,M t)],
                   [(0,CSC t);(0,P t);(0,D t);(1,M t)],(k8,&0))) ]
```

where the function `tumor_growth_model` takes the concentrations of the cell subtypes `CSC`, `P`, `D` and `M` along with eight kinetic reaction rate constants `k1, k2, k3, k4, k5, k6, k7, k8` and real-valued time `t` and returns the corresponding list of bio-chemical reactions involved in the overall tumor growth model.

Our next step is to formally verify the time evolution expressions for `CSC`, `P` and `D` that satisfy the general kinetic model. We formally represent this requirement in the following important theorem:

Theorem 6.2. Time Evolution Verification of Tumor Growth Model

```
⊢ ∀ k1 k2 k3 k4 k5 k6 k7 CSC P D M t k8.
  A1: ~((--k1 + k3 + k4 - k5 + k6 - k7)
            ∗ (--k1 + k3 + k6 - k8)
            ∗ (--k4 + k5 + k7 - k8) = &0) ∧
  A2: ~(k1 - k3 - k4 + k5 - k6 + k7 = &0) ∧
  A3: (∀ t.  CSC t = exp ((k1 - k3 - k6) ∗ t)) ∧
  A4: (∀ t.  P t = ((exp ((k1 - k3 - k6) ∗ t)
            - exp ((k4 - k5 - k7) ∗ t)) ∗ (k2 + &2 ∗ k3)) /
            (k1 - k3 - k4 + k5 - k6 + k7)) ∧
  A5: (∀ t.  D t = (&2 ∗ exp (--k8 ∗ t) ∗ (k2 + &2 ∗ k3) ∗ k5 ∗
            ((-- &1 + exp ((k4 - k5 - k7 + k8) ∗ t)) ∗
            k1 + k3 + k4 - k5 + k6 - k7 +
            exp ((k1 - k3 - k6 + k8) ∗ t)
            ∗ (--k4 + k5 + k7 - k8) +
            exp ((k4 - k5 - k7 + k8) ∗ t)
            ∗ (--k3 - k6 + k8))) /
            ((--k1 + k3 + k4 - k5 + k6 - k7) ∗
            (--k1 + k3 + k6 - k8) ∗ (--k4 + k5 + k7 - k8))) ∧
  A6: real_derivative M t = k6 ∗ CSC t + k7 ∗ P t + k8 ∗ D t
  ⇒ entities_deriv_vec [CSC; P; D; M] t =
    transp (st_matrix (tumor_growth_model CSC P D M k1 k2 k3 k4 k5 k6 k7 k8 t)) ∗∗
    flux (tumor_growth_model CSC P D M k1 k2 k3 k4 k5 k6 k7 k8 t)
```

where the first two assumptions (A1-A2) ensure that the time evolution expressions of `P` and `D` do not contain any singularity (i.e., the value at the expression becomes undefined). The next three assumptions (A3-A5) provide the time evolution expressions for `CSC`, `P` and `D`. The last assumption (A6) is provided to discharge the subgoal characterizing the time-evolution of `M` (dead cells), which is of no interest and does not impact the overall analysis as confirmed by experimental evidences [39]. Finally, the conclusion of the Theorem 6.2 is the equivalent reaction kinetic model of the CSC based tumor growth model.

We start the proof process by rewriting the definitions of `entities_deriv_vec`, `st_matrix` and `flux` in the conclusion of Theorem 6.2. After simplifying the subgoal using the tactic `KINETIC_SIMP` alongside the substitution of the transpose of the 8×4 matrix, we reach the following subgoal:

```
real_derivative CSC t = (k1 - k3 - k6) ∗ CSC t ∧
real_derivative P t = (k2 + &2 ∗ k3) ∗ CSC t + (k4 - k5 - k7) ∗ P t ∧
real_derivative D t = &2 ∗ k5 ∗ P t - k8 ∗ D t
```

We verify the above subgoal with the help of three lemmas involving the real-derivative of `CSC`, `P` and `D`. Here, we only mention one of them which is quite complex and critical in the verification (more details can be found in the source code [36]).

Lemma 6.1. Real Derivative Lemma

```
⊢ ∀ k1 k2 k3 k4 k5 k6 k7 k8 t.
  A1: ~((--k1 + k3 + k4 - k5 + k6 - k7) ∗ (--k1 + k3 + k6 - k8) ∗ (--k4 + k5 + k7 - k8) = &0) ∧
  A2: ~(k1 - k3 - k4 + k5 - k6 + k7 = &0) ⇒
  real_derivative (λ t.  (&2 * exp (--k8 ∗ t) ∗ (k2 + &2 ∗ k3) ∗ k5 ∗ ((-- &1 +
                  exp ((k4 - k5 - k7 + k8) ∗ t)) ∗ k1 + k3 + k4 - k5 + k6 - k7 +
                  exp ((k1 - k3 - k6 + k8) ∗ t) ∗ (--k4 + k5 + k7 - k8) +
                  exp ((k4 - k5 - k7 + k8) ∗ t) ∗ (--k3 - k6 + k8))) /
                  ((--k1 + k3 + k4 - k5 + k6 - k7) ∗ (--k1 + k3 + k6 - k8) ∗
                  (--k4 + k5 + k7 - k8))) t =
  &2 ∗ k5 ∗((exp ((k1 - k3 - k6) ∗ t) - exp ((k4 - k5 - k7) ∗ t)) ∗
  (k2 + &2 ∗ k3)) /(k1 - k3 - k4 + k5 - k6 + k7) -
  k8 ∗ (&2 ∗ exp (--k8 ∗ t) ∗ (k2 + &2 ∗ k3) ∗ k5 ∗
  ((-- &1 + exp ((k4 - k5 - k7 + k8) ∗ t)) ∗ k1 + k3 + k4 - k5 + k6 - k7 +
  exp ((k1 - k3 - k6 + k8) ∗ t) ∗ (--k4 + k5 + k7 - k8) +
  exp ((k4 - k5 - k7 + k8) ∗ t) ∗ (--k3 - k6 + k8))) /
  ((--k1 + k3 + k4 - k5 + k6 - k7) ∗ (--k1 + k3 + k6 - k8) ∗ (--k4 + k5 + k7 - k8))
```

The formal verification of the time-evolution of tumor cell types CSC, P and D in Theorem 6.2 can easily be used to derive the total population and volume of tumor cells using Equations (7) and (8). The derived time-evolution expression can also be used to understand how the overall tumor growth model works. Moreover, the potential drugs are usually designed using the variation of the kinetic rate constants to achieve the desired behavior of the overall tumor growth model. On similar lines, the variation of these parameters are used to plan efficient therapeutic strategies for cancer patients.

6.3 Combined Zsyntax and Reaction Kinetic based Formal Analysis of the Tumor Growth Model

In this section, we consider another model for the growth of tumor cells and formally analyze it using both of our Zsyntax and Reaction kinetics formalizations, presented in Sections 4 and 5 of the report.

6.3.1 Pathway Leading to Death of CSC

The pathway leading to death of CSC is shown in Figure 8. The green-colored circle represents the desired product, whereas, the blued-colored circles describe the chemical interactions in the pathway.

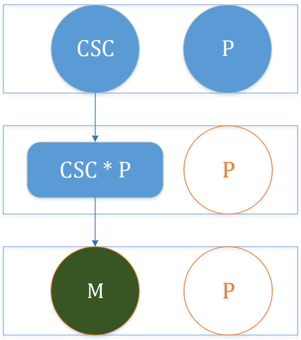

Figure 8: Reaction Representing the Pathway Leading to CSC Death

We use our formalization of Zsyntax to deduce this pathway. In the iclassical Zsyntax format, the reaction of the pathway leading from CSC to its death can be represented by a theorem as CSC & P ⊢ M. Based on our formalization, it can be defined as follows:

Theorem 6.3. The Reaction of the Pathway Leading from CSC to its Death (M)
⊢ DISTINCT [CSC; P; M] ⟹
(zsyn_conjun_elimin (zsyn_deduct [[CSC]; [P]]
[[CSC], [[CSC; P]];
[CSC; P], [[M]]]) [M] = [[M]]

In the above theorem, the first list argument of the function zsyn_deduct represents the IA of molecules that are present at the start of the reaction, whereas the second argument is the list of valid EVFs for this reaction specified in the form of pairs and include the molecules (CSC, P, etc.). We use the HOL Light function DISTINCT to ensure that all molecule variables (from IA and EVFs) used in this theorem represent distinct molecules. Thus, the final list of molecules are deduced under these particular conditions using the function zsyn_deduct. Finally, if the molecule M is present in the post-reaction list of molecules, it will be obtained after the application of the function zsyn_conjun_elimin. We use the simplifier Z_SYNTAX_SIMP [36] to formally verify Theorem 6.3 automatically.

6.3.2 Reaction Kinetic based Formal Analysis of a Tumor Growth based on CSC

We perform the reaction kinetic based formal analysis of a tumor growth model, which is shown in Fig 9. In this model, two types of events are considered: 1) maturation of CSCs into P cells; 2) death of all cell subtypes. All of these types of reactions are driven by different rate constants as shown in Fig 9.

In the following, we provide the possible reactions in the considered tumor growth model:

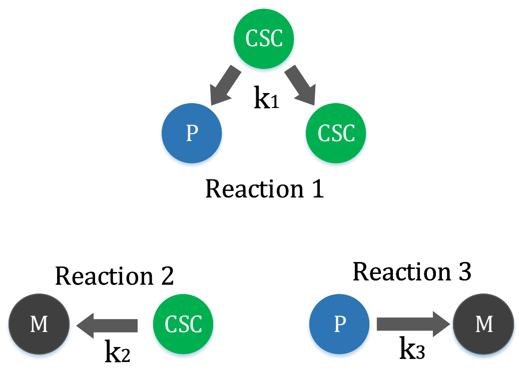

Figure 9: Reaction Representing the Pathway Leading to CSC Death

1. A CSC can undergo asymmetric division (whereby one CSC gives rise to another CSC and a more differentiated P cell), i.e., $\mathrm{CSC} \xrightarrow{k_1} \mathrm{CSC} + \mathrm{P}$.

2. All cellular subtypes can undergo cell death (M), i.e., $\mathrm{CSC} \xrightarrow{k_2} \mathrm{M}$, $\mathrm{P} \xrightarrow{k_3} \mathrm{M}$.

In order to reduce the complexity of the resulting model, only two subtypes of cells are considered: CSCs and transit amplifying progenitor cells (P) as shown in Fig 9. Our main objective is to derive the mathematical expressions, which characterize the time evolution of CSC and P. Concretely, the values of these cells should satisfy the set of differential equations that arise in the kinetic model of the proposed tumor growth. Once the expressions of all cell types are known, the total number of tumor cells (N) in the human body can be computed by the formula as given below:

$$N(t) = CSC(t) + P(t) \tag{9}$$

We formalize the tumor growth model in HOL Light as follows:

Definition 6.2. A Tumor Growth Model

```
⊢ ∀ CSC P M k1 k2 k3 t.
  tumor_growth_rk_model CSC P M k1 k2 k3 t =
   [(irreversible,([(1,CSC t);(0,P t);(0,M t)],[(1,CSC t);(1,P t);;(0,M t)],(k1,&0))) ;
    (irreversible,([(1,CSC t);(0,P t);(0,M t)],[(0,CSC t);(0,P t);(1,M t)],(k2,&0))) ;
    (irreversible,([(0,CSC t);(1,P t);(0,M t)],[(0,CSC t);(0,P t);(1,M t)],(k3,&0)))]
```

where the function `tumor_growth_rk_model` takes the concentrations of the cell subtypes `CSC`, `P` and `M` along with three kinetic reaction rate constants `k1, k2, k3` and real-valued time `t` and returns the corresponding list of bio-chemical reactions involved in the overall tumor growth model.

Our next step is to formally verify the time evolution expressions for `CSC` and `P` that satisfy the general kinetic model. We formally represent this requirement in the following important theorem:

Theorem 6.4. Time Evolution Verification of a Tumor Growth Model

```
⊢ ∀ k1 k2 k3 CSC P M t.
  A1: ~(k3 - k2 = &0) ∧
  A2: (∀t. CSC t = exp (--k2 * t)) ∧
  A3: (∀t. P t = ((k3 - k2 - k1) * exp (--k3 * t) + k1 * exp (--k2 * t)) / (k3 - k2)) ∧
    A4: real_derivative M t = k2 * CSC t + k3 * P t
  ⇒ entities_deriv_vec [CSC; P; M] t =
      transp (st_matrix (tumor_growth_rk_model CSC P M k1 k2 k3 t))
             ** flux (tumor_growth_rk_model CSC P M k1 k2 k3 t))
```

where the first assumption (A1) ensures that the time evolution expression of `P` does not contain any singularity. The next two assumptions (A2-A3) provide the time evolution expressions for `CSC` and `P`, respectively. The last assumption (A4) is provided to discharge the subgoal characterizing the time-evolution of `M` (dead cells), which

is of no interest and does not impact the overall analysis as confirmed by experimental evidences [39]. Finally, the conclusion of Theorem 6.4 is the equivalent reaction kinetic (ODE) model of the CSC based tumor growth model.

We start the proof process by rewriting the definitions of `entities_deriv_vec`, `st_matrix` and `flux` in the conclusion of Theorem 6.4. After simplifying the subgoal using the tactic `KINETIC_SIMP` alongside the substitution of the transpose of the 3×3 matrix, we reach the following subgoal:

```
real_derivative CSC t = --k2 * CSC t ∧
real_derivative P t = k1 * CSC t - k3 * P t
```

We verify the above subgoal with the help of two lemmas involving the real-derivative of `CSC` and `P`. Here, we only mention one of them which is quite complex and critical in the verification (more details can be found in the source code [36]).

Lemma 6.2. Real Derivative Lemma

```
⊢ ∀ k1 k2 k3 t.
  A: ~(k3 - k2 = &0) ⇒
  real_derivative (λ t.  ((k3 - k2 - k1) * exp (--k3 * t) + k1 * exp (--k2 * t)) / (k3 - k2)) t =
                  k1 * exp (--k2 * t) - k3 *
                  ((k3 - k2 - k1) * exp (--k3 * t) + k1 * exp (--k2 * t)) / (k3 - k2)
```

The formal verification of the time-evolution of tumor cell types `CSC` and `P` in Theorem 6.4 can easily be used to derive the total population using Equation 9. The derived time-evolution expression can also be used to understand how the overall tumor growth model works.

This completes our formal verification of the three real-world case studies within the sound core of a theorem prover. The distinguishing feature of our framework is the ability to deductively reason about biological systems using both Zsyntax and reaction kinetics, which has become the norm in modern biological studies. Even though the reasoning in a higher-order-logic theorem prover requires significant user interaction, but we have spent efforts to provide the effective automation where possible. For example, the reasoning about Zsyntax based analysis is automatic as the user does not need to think about the proof steps and which EVFs to apply. Similarly, we developed a simplifier, called `KINETIC_SIMP`, for the reaction kinetics based analysis to reduce the manual reasoning interaction with the theorem prover. However, the most useful benefit of the proposed approach is its accuracy as the theorems are being verified in a formal way using a sound theorem prover. Thus, there is no risk of human error or wrong application of EVFs or simplifications. Finally, due to the computer-based analysis, the proposed approach is much more scalable than the paper-and-pencil based analysis presented in [14, 39]. To the best of our knowledge, our work is the first one to leverage theorem proving to formalize reaction kinetics and Zsyntax which can be considered as a significant step forward towards a long standing goal of applying formal methods in general and theorem proving in particular to various disciplines of science and engineering. Our source code is available for download at [36], and can be used by other biologists and computer scientists for further applications and experimentation.

7 Conclusions

Most of the existing formal verification research related to molecular biology has been focussed on using model checking. However, the inherent state-space explosion problem of model checking limits the scope of this success only to systems where the biological entities can acquire a small set of possible levels. Moreover, the underlying differential equations describing the reaction kinetics are solved using numerical approaches [40], which compromises the precision of the analysis. As a complementary approach, the primary focus of the current report is on using a theorem prover for reasoning about system biology. To this aim, we have developed a framework based on the Zsyntax and reaction kinetics to formally reason about system biology. We consider the concentration of the species of the biological systems as a continuous variable, whereas, that considered in [40] was a discrete, abstract variable. We formally verified properties of Zsyntax and the reaction kinetics of commonly used biological pathways. The practical utilization and effectiveness of the proposed development has been shown by presenting the automatic analysis of the reactions representing the TP53 phosphorylation, death of CSC and reaction kinetics based analysis of tumor growth models.

In future, we plan to conduct the sensitivity and steady state analysis [24] of biological networks that is mainly based on reaction kinetics. We also plan to integrate Laplace [41] and Fourier [42] transforms formalization in our framework that can assist in finding analytical solutions of the complicated ODEs.

References

[1] U. Alon. *An Introduction to Systems Biology: Design Principles of Biological Circuits.* Chapman & Hall/CRC Mathematical and Computational Biology. Taylor & Francis, 2006.

[2] Edwin Wang. *Cancer Systems Biology.* CRC Press, 2010.

[3] Gilles Bernot, Franck Cassez, Jean-Paul Comet, Franck Delaplace, Céline Müller, and Olivier Roux. Semantics of Biological Regulatory Networks. *Electronic Notes in Theoretical Computer Science*, 180(3):3–14, 2007.

[4] C.J. Langmead. Generalized Queries and Bayesian Statistical Model Checking in Dynamic Bayesian Networks: Application to Personalized Medicine. In *Proc. International Conference on Computational Systems Bioinformatics*, pages 201–212, 2009.

[5] Nicholas H Hunt, Jacob Golenser, Tailoi Chan-Ling, Sapan Parekh, Caroline Rae, Sarah Potter, Isabelle M Medana, Jenny Miu, and Helen J Ball. Immunopathogenesis of Cerebral Malaria. *International journal for parasitology*, 36(5):569–582, 2006.

[6] K. Hirayama. Genetic Factors Associated with Development of Cerebral Malaria and Fibrotic Schistosomiasis. *Korean J. Parasitol*, 40(4):165–172, 2002.

[7] L. Thomas and R. d' Ari. *Biological Feedback.* CRC Press, USA, 1990.

[8] R. Thomas. *Kinetic Logic: A Boolean Approach to the Analysis of Complex Regulatory Systems*, volume 29 Lecture Notes in Biomathematics. Springer-Verlag, 1979.

[9] Peter J. E. Goss and J. Peccoud. Quantitative Modeling of Stochastic Systems in Molecular Biology by using Stochastic Petri Nets. *Proceedings of the National Academy of Sciences*, 95(12):6750–6755, 1998.

[10] C. Baier and J. Katoen. *Principles of Model Checking.* MIT Press, 2008.

[11] J. Pospchal and V. Kvasnika. Reaction Graphs and a Construction of Reaction Networks. *Theoretica Chimica Acta*, 76(6):423–435, 1990.

[12] Fabien Corblin, Eric Fanchon, and Laurent Trilling. Applications of a Formal Approach to Decipher Discrete Genetic Networks. *BMC bioinformatics*, 11(1):385, 2010.

[13] M. Magnin L. Paulevé and O. Roux. Abstract Interpretation of Dynamics of Biological Regulatory Networks. *Electronic Notes Theoretical Computer Science*, 272(0):43–56, 2011.

[14] G. Boniolo, M. D'Agostino, and P. Di Fiore. Zsyntax: a Formal Language for Molecular Biology with Projected Applications in Text Mining and Biological Prediction. *PloS ONE*, 5(3):e9511–1–e9511–12, 2010.

[15] R. Pelánek. Fighting State Space Explosion: Review and Evaluation. In *Formal Methods for Industrial Critical Systems*, volume 5596 of *LNCS*, pages 37–52. Springer, 2008.

[16] J. Harrison. *Handbook of Practical Logic and Automated Reasoning.* Cambridge University Press, 2009.

[17] J.H. Woodger, A. Tarski, and W.F. Floyd. *The Axiomatic Method in Biology.* The University Press, 1937.

[18] A. Zanardo and M. Rizzotti. Axiomatization of Genetics 2. Formal Development. *Journal of Theoretical Biology*, 118(2):145–152, 1986.

[19] M. Rizzotti and A. Zanardo. Axiomatization of Genetics. 1. Biological Meaning. *Journal of Theoretical Biology*, 118(1):61–71, 1986.

[20] V. Danos and C. Laneve. Formal Molecular Biology. *Theoretical Computer Science*, 325(1):69–110, 2004.

[21] A. Regev and E. Shapiro. The π-Calculus as an Abstraction for Biomolecular Systems. In *Modelling in Molecular Biology*, Natural Computing Series, pages 219–266. Springer, 2004.

[22] C. Talcott. Symbolic Modeling of Signal Transduction in Pathway Logic. In *Conference on Winter Simulation*, pages 1656–1665, 2006.

[23] W. Fontana. Systems Biology, Models, and Concurrency. *SIGPLAN Notices*, 43(1):1–2, January 2008.

[24] B. P. Ingalls. *Mathematical Modeling in Systems Biology: An Introduction.* MIT press, 2013.

[25] S. Ahmad, O. Hasan, U. Siddique, and S. Tahar. Formalization of Zsyntax to Reason About Molecular Pathways in HOL4. In *Formal Methods: Foundations and Applications*, volume 8941 of *LNCS*, pages 32–47. Springer, 2015.

[26] Konrad Slind and Michael Norrish. A Brief Overview of HOL4. In *Theorem Proving in Higher Order Logics*, pages 28–32. Springer, 2008.

[27] Sohaib Ahmad, Osman Hasan, and Umair Siddique. On the Formalization of Zsyntax with Applications in Molecular Biology. *Scalable Computing: Practice and Experience*, 16(1), 2015.

[28] S. Ahmad, O. Hasan, and U. Siddique. Towards Formal Reasoning about Molecular Pathways in HOL. In *International Conference on Enabling Technologies: Infrastructure for Collaborative Enterprises*, pages 378–383. IEEE, 2014.

[29] J. Harrison. HOL Light: A Tutorial Introduction. In *Formal Methods in Computer-Aided Design*, volume 1166 of *LNCS*, pages 265–269. Springer, 1996.

[30] Michael J Pilling, Paul W Seakins, et al. *Reaction kinetics.* Oxford University Press, 1996.

[31] Sepinoud Azimi, Bogdan Iancu, and Ion Petre. Reaction System Models for the Heat Shock Response. *Fundamenta Informaticae*, 131(3):299–312, 2014.

[32] N. Shankar D. Cyrluk, S. Rajan and M. K. Srivas. Effective Theorem Proving for Hardware Verification. In *Theorem Provers in Circuit Design*, pages 203–222. Springer, 1995.

[33] M. Gordon A. Camilleri and T. F. Melham. *Hardware Verification Using Higher-Order Logic.* University of Cambridge, Computer Laboratory, 1986.

[34] J. M. Schumann. *Automated Theorem Proving in Software Engineering.* Springer Science & Business Media, 2001.

[35] J. Harrison. Formalized Mathematics. Technical Report 36, Turku Centre for Computer Science, Finland, 1996.

[36] A. Rashid. Formal Reasoning about Systems Biology using Theorem Proving - Project's Webpage. `http://save.seecs.nust.edu.pk/projects/sbiology/`, 2015.

[37] O. V. Korobov, V. *Chemical Kinetics with Mathcad and Maple.* Springer, 2011.

[38] Brenton T. Tan, Christopher Y. Park, Laurie E. Ailles, and Irving L. Weissman. The Cancer Stem Cell Hypothesis: A work in progress. *Laboratory Investigation*, aop(current), 2006.

[39] R. Molina-Pena and M. M. Alvarez. A Simple Mathematical Model Based on the Cancer Stem Cell Hypothesis Suggests Kinetic Commonalities in Solid Tumor Growth. *PLoS ONE*, 7(2):e26233, 2010.

[40] Muffy Calder, Vladislav Vyshemirsky, David Gilbert, and Richard Orton. Analysis of Signalling Pathways Using the PRISM Model Checker. In *Proceedings of Computational Methods in Systems Biology (CMSB 2005*, pages 179–190, 2005.

[41] S. H. Taqdees and O. Hasan. Formalization of Laplace Transform Using the Multivariable Calculus Theory of Hol-Light. In *Logic for Programming, Artificial Intelligence, and Reasoning*, pages 744–758. Springer, 2013.

[42] Adnan Rashid and Osman Hasan. On the Formalization of Fourier Transform in Higher-order Logic. In *Interactive Theorem Proving*, volume 9807 of *LNCS*, pages 483–490. Springer, 2016.
